# Immune disease variants modulate gene expression in regulatory CD4^+^ T cells

**DOI:** 10.1016/j.xgen.2022.100117

**Published:** 2022-04-13

**Authors:** Lara Bossini-Castillo, Dafni A. Glinos, Natalia Kunowska, Gosia Golda, Abigail A. Lamikanra, Michaela Spitzer, Blagoje Soskic, Eddie Cano-Gamez, Deborah J. Smyth, Claire Cattermole, Kaur Alasoo, Alice Mann, Kousik Kundu, Anna Lorenc, Nicole Soranzo, Ian Dunham, David J. Roberts, Gosia Trynka

**Affiliations:** 1 Wellcome Sanger Institute, Wellcome Genome Campus, Cambridge, UK; 2 New York Genome Center, New York, NY, USA; 3 NHS Blood and Transplant, Oxford, UK; 4 BRC Haematology Theme, Radcliffe Department of Medicine, University of Oxford, Oxford, UK; 5 European Molecular Biology Laboratory, European Bioinformatics Institute (EMBL-EBI), Wellcome Genome Campus, Hinxton, Cambridge, UK; 6 Open Targets, Wellcome Genome Campus, Cambridge, UK; 7 Institute of Computer Science, University of Tartu, Tartu, Estonia

**Keywords:** regulatory T cell, immune system, transcriptomics, epigenomics, quantitative trait loci, GWAS, immune disease, autoimmunity, expression quantitative trait loci

## Abstract

Identifying cellular functions dysregulated by disease-associated variants could implicate novel pathways for drug targeting or modulation in cell therapies. However, follow-up studies can be challenging if disease-relevant cell types are difficult to sample. Variants associated with immune diseases point toward the role of CD4^+^ regulatory T cells (Treg cells). We mapped genetic regulation (quantitative trait loci [QTL]) of gene expression and chromatin activity in Treg cells, and we identified 133 colocalizing loci with immune disease variants. Colocalizations of immune disease genome-wide association study (GWAS) variants with expression QTLs (eQTLs) controlling the expression of *CD28* and *STAT5A*, involved in Treg cell activation and interleukin-2 (IL-2) signaling, support the contribution of Treg cells to the pathobiology of immune diseases. Finally, we identified seven known drug targets suitable for drug repurposing and suggested 63 targets with drug tractability evidence among the GWAS signals that colocalized with Treg cell QTLs. Our study is the first in-depth characterization of immune disease variant effects on Treg cell gene expression modulation and dysregulation of Treg cell function.

## Highlights

• A dataset of gene expression and chromatin activity in CD4^+^ Treg cells from 124 individuals
• Map of quantitative trait loci (QTLs) for 3,685 genes and 7,195 chromatin regions
• Colocalization of Treg cell QTLs and immune disease GWAS variants to nominate drug targets

## Graphical abstract

**Figure:**
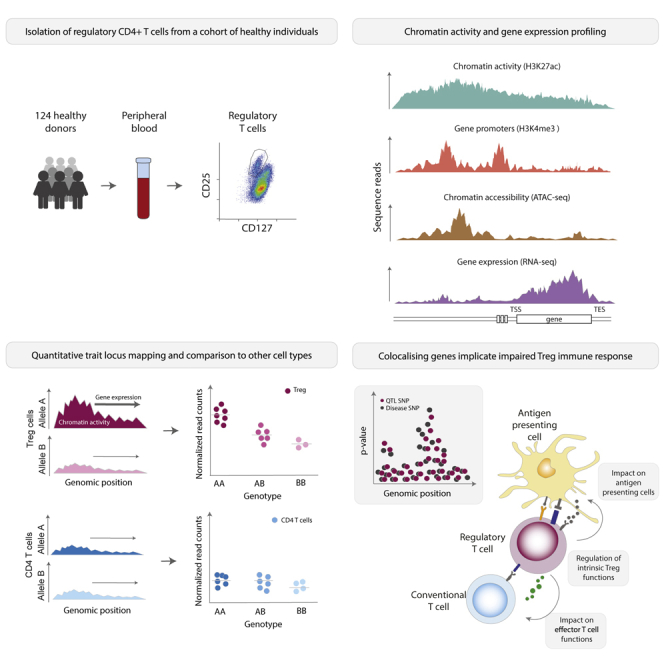

## Teaser

Regulatory T cells (Treg cells) play an essential role in maintaining appropriate immune responses, but their low frequency in circulating blood has resulted in a limited number of available genomic resources. Here, Bossini-Castillo, Glinos, et al. provide a detailed map of gene expression regulation and chromatin activity in Treg cells isolated from 124 healthy individuals.

## Introduction

Thousands of disease variants mapped through genome-wide association studies (GWASs) provide genetic anchors to disease biology, but functional interpretation of GWAS signals has been challenging, as the vast majority of variants are non-coding. One approach for linking genetic variation to downstream effects includes expression quantitative trait locus (eQTL) mapping, in which transcript levels are correlated with genetic polymorphisms.^1^ However, due to the linkage disequilibrium (LD) between genetic variants, the identified eQTLs often result in associations of tens to hundreds of correlated variants with gene expression levels and therefore fail to nominate the causal regulatory variants.

Prioritization of the exact regulatory variants underlying gene expression changes can be further inferred through QTL mapping of chromatin activity using chromatin accessibility or histone modifications (chromatin QTLs [chromQTLs]). In this approach, variants that modulate activity levels of chromatin marks can be physically overlapped with the chromQTL features.^2^ The combination of eQTLs and chromQTLs provides a powerful toolkit for linking non-coding variants to genes whose expression is modulated, for prioritizing functional variants, and for identifying mechanisms through which gene expression is regulated. Finally, colocalization^3^ of disease GWAS signals with such QTLs can point toward causal genes and mechanisms underlying disease associations, therefore linking disease-associated variants to dysregulated pathways and new drug targets.

GWAS variants associated with common immune-mediated diseases, such as inflammatory bowel disease (IBD), type 1 diabetes (T1D), and rheumatoid arthritis (RA), are enriched in active chromatin marks that tag enhancers and promoters in the CD4^+^ T cells, especially in regulatory T cells (Treg cells).^4^, ^5^, ^6^ Treg cells are an infrequent yet functionally significant subset of CD4^+^ T cells; they comprise 2%–10% of CD4^+^ T cells and play an essential homeostatic role in the immune system by suppressing the proliferation and effector functions of conventional T cells. Immunophenotyping studies have shown that abnormal numbers of circulating Treg cells^7^^,^^8^ and defective suppressive function of Treg cells result in a dysregulated immune response in patients with immune diseases,^9^, ^10^, ^11^ as well as in organ and hematopoietic stem cell transplant recipients.^12^^,^^13^ Taken together, the genetic anchor to dysregulation of gene expression in Treg cells and immunophenotyping studies pointing toward impaired function of this cell type strongly suggest that identifying mechanisms through which genetic variants modulate Treg cell function could have important clinical implications. In addition, *ex vivo* approaches to expand Treg cell numbers and to enhance Treg cell suppressive capacity and reinforce them into patients have been successful in clinical trials for T1D^14^, ^15^, ^16^ and Crohn’s disease.^17^

Despite the key role of Treg cells in maintaining appropriate immune responses, their low frequency in circulating blood has resulted in a limited number of available genomic resources.^18^, ^19^, ^20^ Consequently, immune disease variants are often interpreted in light of gene expression data from peripheral blood mononuclear cells (PBMCs), immune cell lines, or isolated major immune cell populations.^2^^,^^21^, ^22^, ^23^, ^24^, ^25^, ^26^ However, these datasets can either dilute or omit gene regulatory effects only present in rare cell types, therefore potentially missing biological effects meaningful to the disease.

Here, to interpret immune disease variants in the context of a cell type strongly relevant to disease biology, we generated the first detailed map of gene expression regulation in Treg cells isolated from 124 healthy individuals. We identified a total of 10,880 QTL effects (3,685 eQTLs and 7,195 chromQTLs). In comparison to closely related naive CD4 T cells as well as monocytes,^24^ we observed 21% of the eQTLs and 29% of the active enhancer and promoter QTLs were detected only in Treg cells. By colocalizing Treg QTLs with variants associated with 14 different immune diseases, we identified 133 GWAS loci with functional relevance in Treg cells. The overlap of immune disease GWAS signals with chromQTLs functionally refined associated variants at 68 immune disease loci. We assigned Treg cell eQTL genes to 81 immune disease loci. At 52 loci, we detected colocalizations with chromQTLs that we were unable to link to downstream gene targets, indicating that the gene regulatory effects could manifest in a cell-state-specific context. Finally, we used the prioritized genes to identify drugs for repurposing and to define novel targets for validation. Our study provides a translational pathway from immune-disease-associated variants, through gene expression regulation in Treg cells, to new treatment options.

## Results

### Comprehensive catalog of gene expression regulation in Treg cells

**Figure 1:**
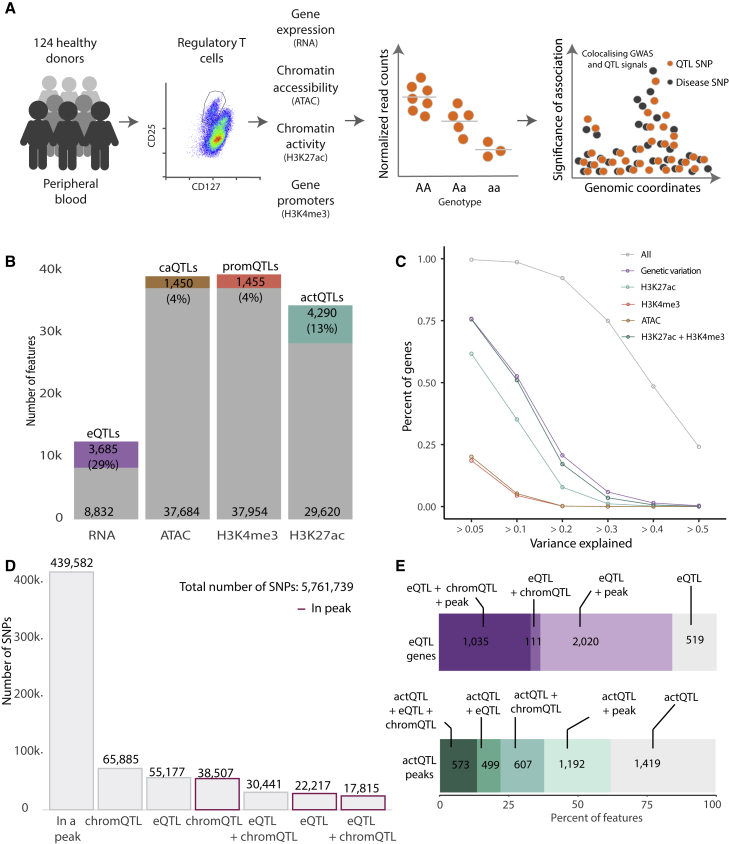
Overview of mapped Treg cell QTLs (A) A schematic of our study design. (B) Number of features defined per genomic assay and number of significant QTLs in each category. (C) Proportion of eQTL gene expression variance explained by genetic variation and chromatin marks. We considered *cis*-regulatory elements in a ±150-kb window from the gene. Shown is the cumulative contribution of genes with increasing proportions of explained variance. (D) Functional classification of tested genetic variants. Bars with purple outline indicate instances when a QTL variant maps to any chromatin peak. Categories are mutually exclusive. (E) Classification of eQTL genes (top) and actQTL peaks (bottom). eQTL genes were classified based on the annotation of eQTL variants with chromQTLs and overlap with chromatin peaks. eQTL + chromQTL + peak, number of eQTL genes for which eQTL variants also result in a chromQTL and one of the eQTL variants mapped within a chromatin mark peak; eQTL + chromQTL, number of eQTL genes for which eQTL variants also result in a chromQTL but no variant mapped within any chromatin mark peak; eQTL + peak, number of eQTL genes for which eQTL variants map within a chromatin mark peak but no chromQTL effects were detected; eQTL, number of eQTL genes for which we were unable to map eQTL variants to chromatin mark peaks or to link them to chromQTLs. actQTL peaks were classified based on the annotation of actQTL variants with eQTLs and overlap with chromatin peaks. actQTL + eQTL + chromQTL, number of actQTLs that also result in an eQTL and an additional chromQTL; actQTL + eQTL, number of actQTLs that also result in an eQTL; actQTL + chromQTL, number of actQTLs that also result in an additional chromQTL; actQTL + peak, number of actQTLs that map within a chromatin mark peak without an additional chromQTL or eQTL; actQTL, number of actQTLs that we were unable to map variants to chromatin mark peaks or to link them to an additional chromQTL or eQTL.

To identify genetic variants that control gene expression regulation in Treg cells isolated from healthy blood donors (Figure S1; Table S1), we profiled the transcriptome using RNA sequencing (RNA-seq) (124 individuals), chromatin accessibility using assay for transposase-accessible chromatin using sequencing (ATAC-seq) (73 individuals), promoters using H3K4me3 (88 individuals), and active enhancer and promoter regions using H3K27ac (91 individuals; Figure 1A). We detected the expression of 12,517 genes, while chromatin profiling revealed 39,134 accessible regions, 39,409 H3K4me3 marked promoter regions, and 33,910 H3K27ac marked active chromatin regions (Figures 1B and S2). The majority of the mapped regulatory chromatin features overlapped with each other (Figure S2A). Concordant with previous studies,^24^^,^^27^^,^^28^ we observed that H3K4me3 and chromatin accessible regions were concentrated near the transcription start sites (TSSs), while H3K27ac marked more distal gene-regulatory elements (Figure S2B). Concordantly with previously described DNA acetylation patterns, the H3K27ac peaks were wider than H3K4me3 and ATAC peaks (Figure S2C). Moreover, the fraction of reads in peaks and the correlation between replicates confirmed the quality of the defined features (Figures S2D and S2E).

Using the 62 samples for which we had complete information, including genetic variation, chromatin profiles, and whole transcriptome, we estimated the percentage of gene expression variability explained by the genetic component and by the chromatin regulatory features. We observed that the major component driving transcriptional variability was the common genetic variation; for 75% of the eQTL genes, we were able to explain 5% or more of the expression variance (Figure 1C). With the addition of the combination of chromatin marks (H3K27ac, H3K4me3, and ATAC) and the common genetic variation, we were able to explain 5% or more of the gene expression variance for all the eQTL genes. This additional gene expression variability was mainly accounted for by the combination of H3K27ac and H3K4me3. These results were in line with previous reports for other primary immune cells.^24^ Together, the gene expression variance decomposition analysis implicated that genetic variation contributed the most toward gene expression regulation and the genetic regulation was present at both the transcriptome and the chromatin mark levels. Therefore, by connecting genetic variation to gene expression and chromatin regulatory features, we expected our Treg cell dataset to provide translational insight into immune disease GWAS loci.

Next, we performed QTL mapping to define genes and chromatin features that were under genetic control in Treg cells (STAR Methods). We detected at least one independent association for 3,685 genes (29%) and a total of 125,650 eQTL variants (eQTLs) (Figures 1B–1D; Table S2). We mapped a total of 7,195 chromQTLs, using chromatin accessibility (caQTLs, 1,450; 4%), H3K4me3 (promQTLs, 1,455; 4%), and H3K27ac (actQTLs, 4,290; 13%) histone marks, which corresponded to 9,292 non-overlapping peak regions, associated with 152,648 chromQTL variants. The majority of chromQTLs were detected in H3K27ac features (4,290 actQTLs; Figure 1B). Of all analyzed genetic variants (5,761,739), 439,582 (7%) fall within a peak; however, only a small fraction mapped in a chromatin feature and were also linked to chromQTLs (38,507 SNPs; 0.7%), eQTLs (22,217 SNPs; 0.4%), or both (17,815 SNPs; 0.3%; Figure 1D). For 28% (1,035) of all eQTL genes, we observed that at least one eQTL variant was a chromQTL and was also physically located in a chromatin peak (Figure 1E), and for an additional 2,020 eQTL genes, we were able to link an eQTL variant to a chromatin peak, though without detecting a QTL effect on a chromatin feature. A proportion of this overlap may not be functional, as chromatin regulatory features are abundant throughout the genome and therefore likely to overlap common genetic variants by chance. Interestingly, we were unable to link the majority of actQTL variants to an eQTL (Figure 1E), implicating that these regulatory regions may modulate gene expression under a specific cellular context or through the interplay of multiple regulatory elements.

### Defining gene expression regulation in Treg cells

We sought to identify QTL effects at the levels of gene expression and chromatin regulation specific to our Treg cell dataset and absent from other immune cells assayed in publicly available data. However, we recognize that such a comparison can suffer from confounders introduced by technical biases, such as differences in sample processing. Therefore, we used transcriptomics data from 91 individuals sampled by the Database of Immune Cell eQTLs Expression Epigenomics (DICE) consortium,^18^ where different immune cell types were assayed from the same donors (STAR Methods). We retrieved data for naive T cells and memory Treg cells to directly estimate the proportion of replicable eQTL effects with our data. As a comparison, we included classical monocytes, as we expected the degree of sharing to be lower compared with Treg cells. We used pairwise pi1 score,^29^ which estimates the proportion of true positive associations replicating between discovery and replication cohorts. Indeed, we observed that the eQTLs detected in our Treg cell cohort replicated highly in memory Treg cells (pi1 = 0.85) and naive T cells (pi = 0.84) in the DICE data, while the sharing was lower in monocytes (pi1 = 0.71; Figures S3A and S3B). The eQTLs detected in the DICE Treg cell cohort replicated more highly in our Treg cell dataset and also replicated more highly compared with DICE naive T cells, meaning that we replicated the majority of DICE eQTLs, which is likely due to differences in sample size and the average sequencing depth being greater in our dataset (Figure S3B).

**Figure 2:**
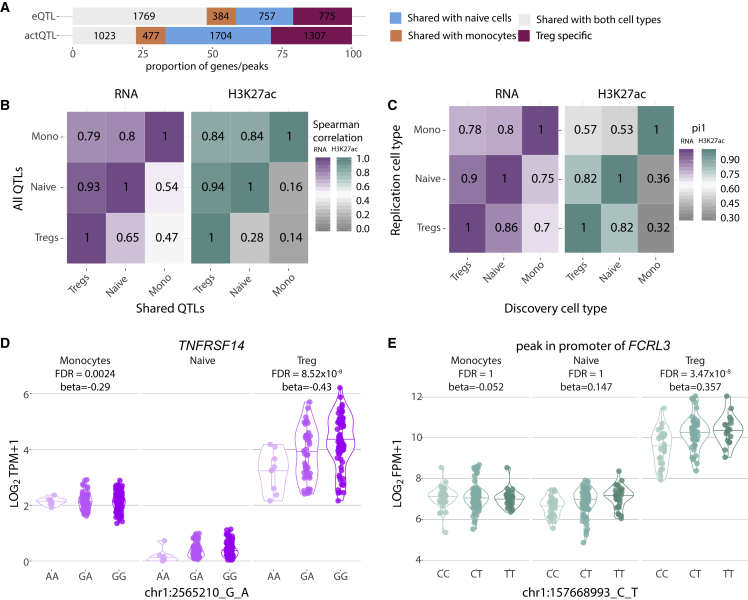
Comparison of eQTLs and actQTLs identified in regulatory T cells, CD4^+^ naive cells, and monocytes (A) Proportion of eQTLs and actQTLs specific to Treg cells in comparison to naive T cells and monocytes. (B) Pairwise pi1 score between the three cell types for eQTLs and actQTLs. (C) Spearman correlation between the regression slopes for the same gene or peak and variant pairs of all eQTLs and actQTLs (colored) and only for the shared pairs (gray). (D and E) Examples of Treg-cell-specific (D) eQTLs and (E) actQTLs. FPM, fragments per million; TPM, transcripts per million. FDR, false discovery rate.

Having confirmed that our dataset was capturing effects relevant to Treg cell biology, we next used the CD4 naive T cells from BLUEPRINT project,^24^ as it profiled both the transcriptome and H3K27ac assayed across a similar cohort to ours (197 British healthy individuals). Again, we included monocytes, as we expected lower sharing compared with naive CD4^+^ T cells. We observed that the majority of eQTLs (69%; Figure 2A; Table S2) were shared with naive CD4 T cells, with similar effect sizes and the same direction of effects (Figures S4A and S4B). A higher correlation between eQTL effects was observed between Treg cells and naive T cells (Spearman R^2^ = 0.93) than between Treg cells and monocytes (Spearman R^2^ = 0.76), also confirmed by the pi1 estimates (Figures 2B and 2C). Despite the substantial eQTL sharing between Treg cells and the other two cell types, we classified 775 genes (21% of all eQTL genes) as specific to our Treg cell dataset, including 92 genes that were only expressed in Treg cells (intersection between 187 genes only expressed in naive T cells and 384 genes only expressed in monocytes; STAR Methods; Figures 2A and S4B). Of these 775 genes, 695 were also only detected in Treg cells compared with the naive T cells and monocytes from the DICE consortium, while of the 92 genes expressed only in Treg cells, 82 were also only expressed in Treg cells compared with the DICE consortium (Figure S4C). eQTL genes specifically expressed in Treg cells, but not in the other two cell types, showed lower expression levels compared with genes expressed in all cell types (Figure S4D). Therefore, some of the eQTL effects could be shared with the other cell types and the higher sequencing depth of our study enabled capturing the transcripts of these genes while they were not detected in the BLUEPRINT datasets. Among the Treg-cell-specific eQTLs, there were many genes essential to immune function regulation, including *TNFRSF14* (false discovery rate [FDR] = 2.86 × 10^−4^), a chemokine that attracts lymphocytes toward epithelial cells (Figure 2D). A *TNFRSF14* eQTL is also found in monocytes, but the variants are in low LD, while in naive T cells, the gene is expressed at very low levels.

To compare the genetic effects across the same peak regions in all three cell types, we performed peak calling on reads merged from all cell types (see STAR Methods). When we compared the actQTLs across the three cell types, we observed that 1,307 (29%) actQTLs were Treg-cell-specific (Figures 2A and S4B). Although the concordance of the effect sizes across all peaks was small (Spearman R^2^ ≤ 0.28), peaks with shared QTLs expressed similar effect sizes (R^2^ ≥ 0.84; Figure 2B). As expected, there was a higher correlation of effect sizes between Treg cells and naive T cells (Spearman R^2^ = 0.94) than between Treg cells and monocytes (Spearman R^2^ = 0.79; Figure S4A). Results from the pi1 analysis also reflected our observations from the RNA that the replication of Treg cell QTLs was higher in the naive dataset compared with the monocyte (Figure 2C). Among the Treg-cell-specific actQTL effects, we observed a peak in the promoter of *FCRL3* gene (chr1:157,693,404–157,705,914; FDR = 3.47 × 10^−8^) that is a potential negative regulator of Treg cell suppressive function (Figure 2E).^30^

### Treg cell QTLs colocalize with immune-disease loci

**Figure 3:**
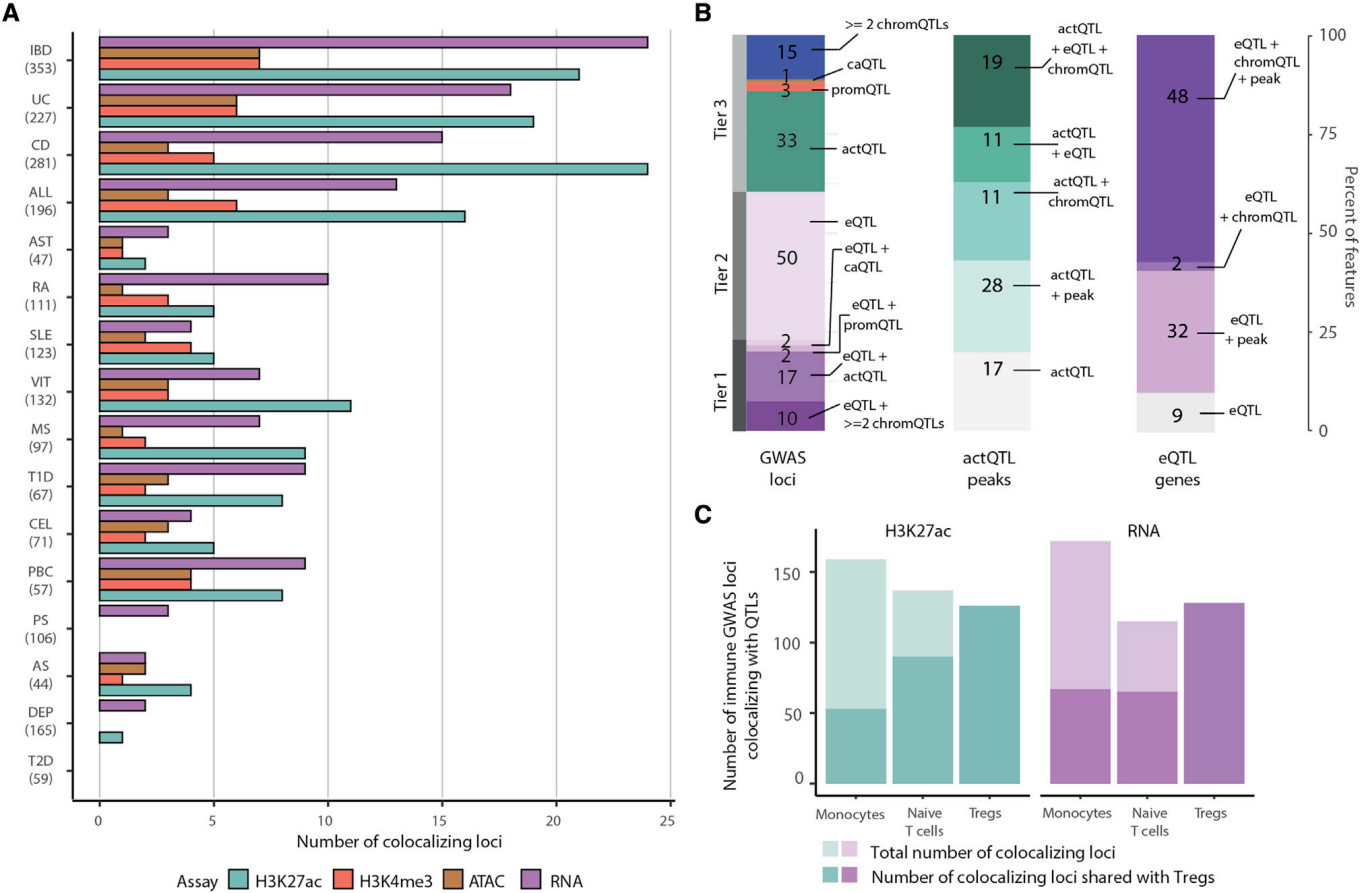
Colocalization of immune disease GWAS loci and Treg cell QTLs (A) Distribution of Treg cell eQTLs and chromatin QTLs colocalizing with different immune disease GWAS loci. Number in parentheses is state-independent loci associated with the trait. The numbers on the right side of the bars correspond to the total number of features (genes or peaks) tested for colocalization. ALL, allergic disease (asthma, hay fever, and eczema); AST, asthma; CD, Crohn’s disease; CEL, celiac disease; DEP, broad depression; IBD, inflammatory bowel disease; MS, multiple sclerosis; PBC, primary biliary cirrhosis; PS, psoriasis; RA, rheumatoid arthritis; SLE, systemic lupus erythematosus; T1D, type 1 diabetes; T2D, type 2 diabetes; UC, ulcerative colitis; VIT, vitiligo. (B) Distribution of the GWAS loci colocalizing with different types of Treg cell QTLs. (C) Number of immune GWAS loci colocalizing with monocyte, naive T cell, and Treg cell eQTLs and actQTLs.

To fine-map disease-associated loci to causal genes and variants, we next integrated the Treg cell QTL results with GWAS signals from common immune diseases. We applied a Bayesian framework to test for statistical colocalization of the disease-associated variants and the Treg cell QTL signals.^31^ Collectively, we tested 1,290 unique GWAS loci associated with 14 immune-mediated diseases: allergic diseases (ALL), ankylosing spondylitis (AS), asthma (AST), celiac disease (CEL), Crohn’s disease (CD), inflammatory bowel disease (IBD), multiple sclerosis (MS), primary biliary cirrhosis (PBC), psoriasis (PS), rheumatoid arthritis (RA), systemic lupus erythematosus (SLE), type 1 diabetes (T1D), ulcerative colitis (UC), and vitiligo (VIT) (see STAR Methods and Table S3). Diseases with the highest number of colocalizations (more than 20 colocalizing signals) included IBD, UC, CD, ALL, T1D, VIT, and PBC (Figures 3A and S5A). The high number of observed colocalizations is consistent with previous work that implicated the role of Treg cells in the pathobiology of all of these diseases^5^^,^^6^^,^^32^, ^33^, ^34^ and to some extent also reflects the greater number of significant GWAS loci for these traits. Overall, immune-mediated diseases showed more colocalizations with Treg cell QTLs than non-immune-mediated diseases, such as type 2 diabetes or depression.

Four of the colocalizing eQTLs were shared between three or more diseases and included *BACH2* (T1D, AS, and MS), *SUOX* (ALL, VIT, and T1D), *TYK2* (PBC, RA, SLE, and T1D), and *ZFP90* (PS, ALL, and UC). The BACH2 locus also contained an actQTL (chr6:90,264,695–90,268,560), which colocalized with ALL, AST, MS, CEL, VIT, CD, and IBD. Similarly, *SUOX* colocalized with an actQTL (chr12:55,989,136–56,011,728), a promQTL (chr12:55,996,308–55,998,877), and a caQTL (chr12:56,041,233–56,042,198) for T1D, ALL, and VIT. We observed the largest number of colocalizations with Treg cell actQTLs. There were also chromQTLs that colocalized with multiple diseases but did not have a corresponding colocalizing eQTL. Among them, we identified (1) a region upstream of *CXCR5*, which colocalized with an actQTL (chr11:118,866,698–118,871,517), a caQTL (chr11:118,869,935–118,870,610), and a promQTL (chr11:118,869,586–118,871,234; CEL, RA, and PBC); (2) an actQTL (chr11:76,586,431–76,600,121) upstream of *LRRC32* (ALL, AST, T1D, UC, CD IBD, and T1D); (3) a promQTL in an intron of *ZMIZ1* (chr10:79,240,389–79,246,577; AS, MS, and IBD); and (4) an actQTL (chr17:39,751,832–39,807,281; SLE, ALL, and PBC) and a promQTL (chr17:39,912,458–39,929,022; PBC, AST, T1D, SLE, and ALL) in the same region but covering *IKZF3* and *ORMDL3*, respectively (Table S3).

We observed 360 significant colocalizations between the disease loci and at least one Treg cell QTL, corresponding to 133 unique GWAS loci (Figure 3B; Table S3). Of the 133 unique GWAS loci, 50 loci colocalized with eQTLs only, 52 with chromQTLs only, and 31 colocalized with both eQTL and at least one chromQTL. The colocalizations with both transcriptomic and chromatin evidence affected the expression of 37 eQTL genes, acetylation of 31 actQTL peaks, methylation of 10 promQTL peaks, and accessibility of 11 caQTL sites. Of the immune disease GWAS loci that colocalized with both Treg cell eQTLs and chromQTLs, 27 out of 31 comprised actQTLs (87%). Finally, for the vast majority (79%) of the loci where we observed disease signals colocalizing with two or more types of QTLs, the effects of the risk alleles propagated in the same direction. For example, the *CCL20* eQTL colocalized with UC variants, tagged by chr2:228,670,575, and the risk allele resulted in both reduced gene expression and decreased H3K27 acetylation (chr2:227,804,673–227,819,6), H3K4 tri-methylation (chr2:227,805,541–227,808,260), and chromatin accessibility (chr2:227,805,505–227,805,928; Table S3). However, at 10 loci, we observed that the disease alleles resulted in opposite effects between the different types of QTLs, suggesting complex mechanisms of gene expression regulation (Table S3).

We systematically investigated all immune disease signals colocalizing with Treg cell QTLs to refine the disease-associated signals to sets of functional variants and to nominate causal genes. We classified colocalizing loci into three categories. Tier 1 loci comprised 31 signals for which the GWAS association colocalized with both eQTL and chromatin QTL (Figure 3B; Table S4). Of these, at 25 loci, the associated variants were also located within the chromQTL peaks. Loci in this category were the most informative to functionally refine disease associations, as we were able to link the GWAS signals to genes and to functional chromatin elements that regulated gene expression. Tier 2 loci contained 50 signals for which we observed colocalization only with eQTLs. In this case, we were unable to refine the association signals to sets of functional variants, but we were able to connect the GWAS signals to candidate causal genes. Finally, the 52 loci in tier 3 included GWAS signals colocalizing with chromatin QTLs, but not eQTLs. Of these, at 40 loci, the GWAS variants overlapped a chromatin QTL peak, providing further clues to prioritize functional variants at GWAS loci (Table S4). Finally, tier 3 loci represented the majority of colocalizations. We hypothesized that gene expression effects could be manifested in a cell-state-specific context. To further nominate candidate genes regulated by the variants colocalizing with actQTLs, we used resting and activated Treg cell transcriptome data (see STAR Methods) and defined genes proximal to the QTL peaks that were differentially expressed upon cell activation (Table S5). This analysis prioritized 124 genes linked to 44 disease-colocalizing actQTLs. We went on to carry out allele-specific expression analysis for these loci and validated 36 of these genes as displaying imbalanced expression with regards to the lead GWAS variant (STAR Methods). In parallel, we used cap analysis of gene expression (CAGE) data from FANTOM5^35^ and linked the enhancer usage of 50 of the disease-colocalizing actQTLs to the TSS expression of 374 genes (STAR Methods). Overlapping these approaches, we found 34 actQTLs connected to 56 genes differentially expressed upon Treg cell stimulation, 23 of which displayed allele-specific expression, including CD247, LRRC32, and PRDM1. For a subset of loci, we have therefore compiled candidate gene target lists based on allelic and gene expression evidence across platforms.

Next, we assessed which of the identified eQTLs that colocalized with immune disease variants regulated gene expression specifically in Treg cells and not in naive T cells or monocytes. Out of the 81 GWAS loci that showed colocalization with Treg cell eQTLs, 31 were Treg cell exclusive and not present in naive T cells or monocytes (Figures 3C, S5C, and S5D). Similarly, 21 of 78 of actQTLs loci were Treg cell exclusive. Three of the Treg-cell-specific colocalizing eQTLs also had specific colocalization with a Treg cell actQTL: *MAP3K8*, which colocalized with UC and IBD; *IFITM1* colocalizing with PBC; and *TLR1* colocalizing with ALL. Treg-cell-colocalizing actQTLs were enriched for JUN, GATA3, and STAT6 transcription factors (Table S6).

### Colocalizing Treg cell QTLs prioritize immune disease causal variants and genes

**Figure 4:**
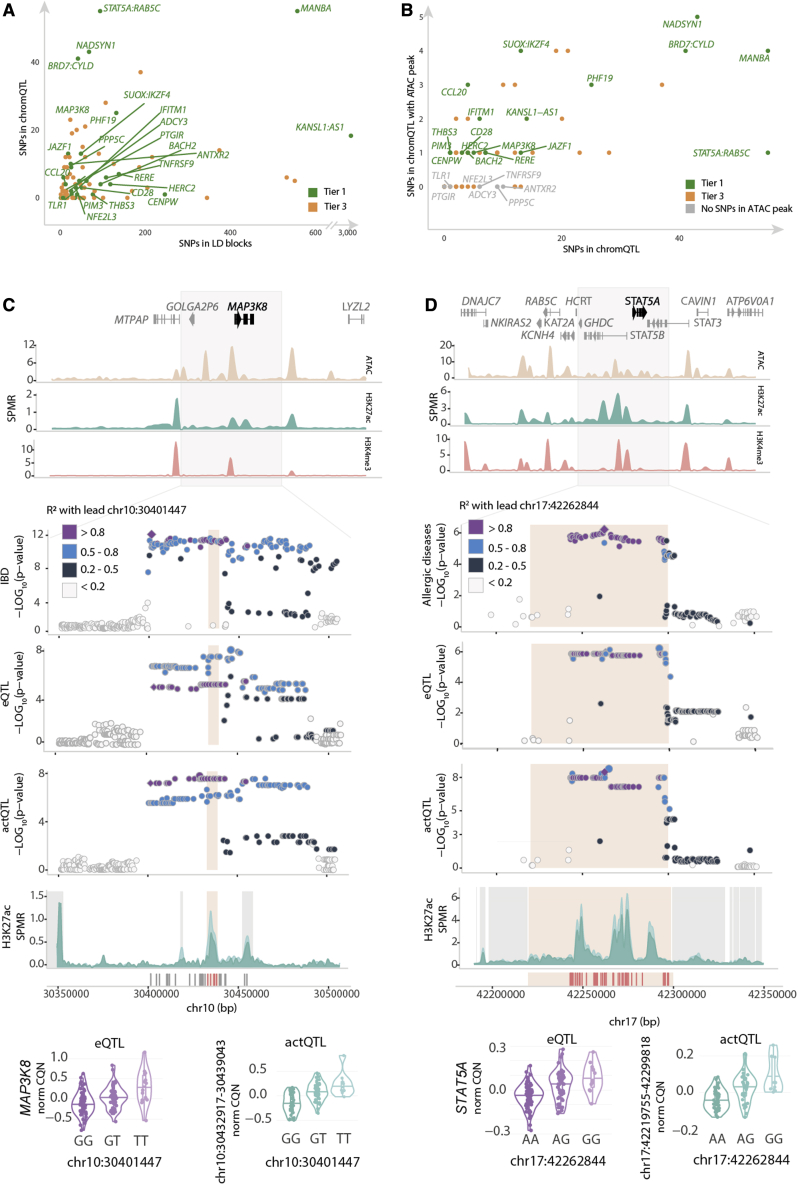
Functional refinement of immune disease associations colocalizing with Treg cell QTLs (A) The number of SNPs in LD blocks (lead GWAS signals and their proxies R2 ≥ 0.8) on the x axis and the number of SNPs that map inside chromQTL peaks on the y axis. (B) The number of SNPs in LD blocks that map inside chromQTL peaks on the x axis and the number of SNPs that map inside both chromQTL and an additional ATAC peak on the y axis. (C and D) From top to bottom, the figure displays gene annotation tracks; chromatin landscape for ATAC-seq, H3K27ac, and H3K4me3 ChM-seqs; region association plots for disease; eQTL and actQTL association p values, focused on H3K27ac landscape stratified by homozygous genotypes; and genotype-stratified eQTL and actQTL violin plots. (C) Locus associated with IBD, tagged by chr10:30,401,447 (rs10826797) SNP colocalizing with MAP3K8 eQTL and chr10:30,432,917–30,439,043 actQTL is shown. (D) Locus associated with allergies, tagged by chr17:42,262,844 (rs7207591) SNP colocalizing with STAT5A eQTL and chr17:42,219,755–42,299,818 actQTL is shown. CQN, conditional quantile normalized reads; SPMR, signal per million reads.

Using the tier 1 and tier 3 loci that overlapped with chromatin QTL peaks, we refined the signals at 68 GWAS loci from a median of 48 associated variants to six functional variants per locus (Figure 4A; Tables S4 and S7). Of the 68 loci, in 45 instances, we observed that the genetic variants additionally overlapped open chromatin peaks, allowing us to further prioritize the functional variants from an average of 13 functional variants to an average of two variants per locus, including *BACH2*, *CD28* (Figure S6), *CENPW*, *HERC2*, *JAZF1*, *MAP3K8*, *PIM3*, *RERE*, *STAT5A*, and *THBS3* loci, which colocalized with eQTLs and were refined to a single functional variant (Figure 4B; Table S4). In the case of previously statistically fine-mapped loci, in which associations have been refined to rare variants or haplotypes, such as *CD28*, *BACH2*, *CTSH*, and *TYK2*,^36^^,^^37^ the information from Treg cell QTL colocalizations prioritized additional functional variants.

Treg-cell-exclusive colocalizations along with the Treg cell actQTL-specific colocalizations indicated regulation of pathways that were characteristic of Treg cell biology. We therefore investigated in more detail the Treg-cell-exclusive colocalization with an IBD GWAS signal, tagged by the chr10:30,401,447 (rs10826797) variant, which colocalized with an actQTL, regulating a 6-kb-large (chr10:30,432,917–30,439,043) H3K27ac peak (p = 9.5 × 10^−9^) at the TSS of *MAP3K8* and an eQTL (p = 9.8 × 10^−6^) for the *MAP3K8* gene (Figure 4C; Table S3). The IBD risk allele decreased the acetylation at H3K27 and downregulated the expression of *MAP3K8*. Five of the colocalizing variants overlapped this actQTL peak, of which only one SNP, chr10:30,434,664 (rs306588), overlapped a 1.5 kb ATAC peak (chr10:30,433,210–30,434,733; Tables S3 and S7). This approach refined the IBD-associated signal from 30 GWAS variants to a single functional candidate variant regulating the expression of *MAP3K8*, a kinase modulating the DNA-binding activity of FoxP3, the Treg cell hallmark transcription factor.^38^

In another example, we observed that a locus associated with allergies^39^ (tagged by the index SNP chr17:42,262,844 [rs7207591]) colocalized with a *STAT5A* eQTL (p = 3.9 × 10^−6^), as well as with an 80-kb actQTL (chr17:42,219,755–42,299,818; p = 4.2 × 10^−9^; Figure 4D). This peak overlapped the *STAT5A* TSS. Nearly half of the LD block of allergy variants (55 out of 93 SNPs) overlapped with the regulated actQTL peak and one of the variants, chr17:42,266,938 (rs34129849), also mapped to a 629-bp open chromatin region (chr17:42,266,595–42,267,224) located in intron one of the *STAT5B* gene (Figure 4D; Table S3). Modulation of STAT5-mediated pathways could implicate broad effects on Treg cell function as STAT5A regulates the expression of genes downstream of the interleukin-2 (IL-2) receptor, which is critical for Treg cell development and function.^40^

### Treg cell QTLs emphasize CD28 co-stimulation, tumor necrosis factor, and IL-10 signaling pathways for drug targeting

**Figure 5:**
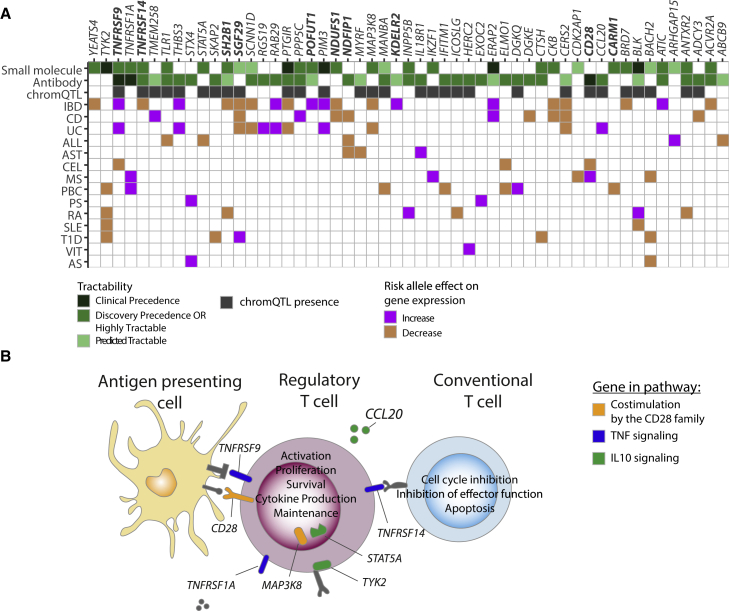
Immune disease colocalizations with Treg cell QTLs inform drug targets (A) Tier 1 and Tier 2 loci colocalizing with immune disease GWAS variants with drug tractability evidence (green). In bold are Treg-cell-specific eQTLs. Clinical precedence, gene targeted by small molecules or antibodies approved for patient treatment or undergoing clinical trials; discovery precedence, gene product shown to bind small molecules; predicted tractable, gene predicted to be small molecule tractable; tractable high confidence, gene product with high predicted tractability as an antibody drug target; tractable medium–low confidence, gene product with predicted tractability as an antibody drug target. NDUFS1 is not directly targeted but is part of a targeted complex. (B) Tier 1 and tier 2 genes with tractability potential in CD28 co-stimulation (orange), TNF (blue), and anti-inflammatory IL-10 (green) pathways.

Despite the success of GWAS in mapping disease risk variants, the efforts to translate these findings into drug targets have been challenging. Therefore, we used the Open Targets Platform^41^ to systematically assess whether eQTLs that colocalized with immune disease signals identify known and potential new drug targets (see STAR Methods). Of the 91 eQTL genes that colocalized with immune diseases and could be tested in the Open Targets Platform, we found nine (tier 1: *BLK*, *CD28*, *PIM3*, *PTGIR*, and *TNFRSF9* and tier 2: *ERAP2*, *NDUFS1*, *TNFRSF1A*, and *TYK2*; Figure 5A) that were already targeted by known drugs and were either used in clinical practice or undergoing clinical trials. Seven of these eQTL genes could be considered for drug repurposing: *ERAP2*, *NDUFS1*, *PIM3*, *PTGIR*, *TNFRSF1A*, *TNFRSF9*, and *TYK2*, three of which are Treg-cell-specific eQTLs. However, most of the drugs targeting these genes are used for cancer therapies, where the desired effects include dampening the suppressive capacity of Treg cells, in contrast to immune diseases where the enhancement of Treg cell function is sought after. Nevertheless, this analysis highlighted some potential drug candidates, for example, a colocalization between a *NDUFS1* eQTL and CD, in which the disease risk allele increased gene expression, suggested repurposing metformin, which targets the NADH dehydrogenase complex (not directly *NDUSF1*). Metformin is currently used for treating type 2 diabetes; in a clinical trial for MS patients, it increased the number of Treg cells,^42^ and in *in vitro* studies, it promoted Treg cell proliferation.^43^

In addition, we observed 63 genes that were not yet a part of a clinical treatment but had drug tractability evidence, of which 47 were classified as highly tractable (eight of which were specific to Treg cells; Figure 5A; Tables S8 and S9). We used Open Target’s definition of tractability (druggability), which is based on availability of a binding site in the protein that can be used for small-molecule binding, presence of an accessible epitope for antibody-based therapy, or reports of a compound in clinical trials with a modality other than small molecule or antibody. An example of a highly tractable gene was *ERAP2*, for which we observed the IBD and CD risk allele colocalizing with the *ERAP2* eQTL increased gene expression, implicating *ERAP2* as a target for validation. Collectively, we observed that genes with high tractability evidence fell into three pathways: co-stimulation by the CD28 family (p = 0.012), tumor necrosis factor (TNF) signaling (p = 0.0034), and IL-10 signaling (p = 0.01; Figure 5B). These pathways play an important role in Treg cell activation, proliferation, and survival, as well as in suppression of effector T cells.

Finally, of the 91 genes that were tested, 44 had been knocked down or knocked out in mice, of which 26 had a reported immune system phenotype (Table S10). Among those, six gene knockouts resulted in an immune disease, including Cd28, Ndfip1, Skap2, Tmem258, Tnfrsf1a, and Tnfrsf9. For example, Tnfrsf1a^−/−^ decreased susceptibility to experimental autoimmune encephalomyelitis, consistent with our observation that the risk allele for multiple sclerosis in Treg cells leads to increased *TNFRSF1A* gene expression levels. In addition, Icosl, Ikzf1, Map3k8, Pofut1, Ptgirgir, and Stat5a had specific organ inflammatory phenotypes, such as reduced small intestine inflammation in Map3k8-deficient mice.^44^ It is important to note that we did not observe the same direction of effects for all mouse knockouts, which could be partly due to the fact that gene perturbations are not Treg cell specific.

## Discussion

Pinpointing genes that are regulated by disease-associated non-coding variants can uncover important cell pathways for drug targeting. However, leveraging information captured by GWAS variants to provide insight into disease biology and improve treatment has been challenging. Increasing availability of functional genomic resources from different cell types helps to bridge this gap. Naive and regulatory CD4^+^ T cells are closely related, yet they play distinct functions in the immune system. Although naive CD4^+^ T cells have been extensively characterized, Treg cells are an infrequent cell population difficult to isolate in large numbers for QTL analysis and elusive to deconvolute from bulk blood QTL data.^45^ Therefore, mapping gene expression regulation directly in Treg cells is essential to better understand Treg cell biology.

Here, we sought to describe the role of immune-disease-associated variants on modulation of gene expression in Treg cells. We linked 133 unique immune disease loci from associated variants to functional effects in Treg cells; 50 loci were linked to gene expression, 52 loci were linked to an effect on chromatin, and 31 loci to both. Loci for which we observed colocalization with both gene expression and chromatin QTLs provide an important translational insight into mechanisms through which immune disease variants regulate Treg cell function. For example, we observed signals overlapping with Treg-cell-specific eQTLs, indicating regulation of essential Treg cell pathways, such as IL-2 signaling via *STAT5A*.

The 52 loci for which we only detected colocalization with chromQTLs, but not eQTLs, indicate that the altered gene expression may be manifested in a specific cell state, which will require tailored functional follow-up studies. In a separate study, we demonstrated that the disease colocalization with actQTL near *LRRC32* (encoding GARP) resulted in reduced GARP expression in activated Treg cells and subsequently led to reduced Treg cell suppression, which promoted development of colitis.^46^ In addition, previous studies showed that context-specific eQTLs can be already detected in a resting state at the chromQTL level.^2^^,^^22^^,^^24^

On the other hand, the 50 loci colocalizing only with eQTL variants, but not chromQTLs, may be correlated with chromatin-independent gene expression regulation, such as splicing QTLs (sQTLs)^47^ or RNA stability.^48^ For example, we observed that *ERAP2*, an IBD- and CD-associated locus, showed an eQTL colocalization but no chromQTL effect. The lead GWAS variant chr5:96,912,106 (rs6873866) and the colocalizing lead eQTL variant chr5:96,916,728 (rs2927608) are proxies for chr5:96,900,192 (rs2248374), a sQTL present in monocyte-derived dendritic cells after influenza infection and type 1 interferon stimulation.^49^

By linking immune disease GWAS variants to Treg cell eQTLs, our study contributes toward building genetic evidence for the causal role of Treg cells in disease biology and supports the discovery and repurposing of drugs that modulate Treg cell function in treating immune disease patients. Validation of targets with genetic support can significantly increase the chance of clinical success.^50^^,^^51^ Our results support the focus on modulating co-stimulatory and cytokine pathways, for example, at a CEL locus, the disease risk alleles led to decreased levels of expression of *TNFRSF9* (encodes for CD137/4-1BB). Signaling via CD137 induces cell division and proliferation;^52^^,^^53^ however, *TNFRSF9* gene expression and protein levels increase specifically in activated Treg cells, but not in conventional T cells.^54^^,^^55^ Furthermore, the increased expression of CD137 enhances the Treg cell capacity to suppress proliferation of effector T cells.^56^ Therefore, the disease risk allele could result in decreased Treg cell suppressive function and promote immune disbalance. The colocalization of several TNF receptor superfamily members (*TNFRSF1A*, *TNFRSF9*, and *TNFRSF14*) further supports the development of drugs modulating TNF pathway, one of the main therapy lines for treating immune diseases.

Understanding the genetic underpinnings of immune system regulation has broad implications not only in the treatment of immune-mediated conditions but also in infections, transplantation, and cancers. For instance, in organ transplantation, numbers of Treg cells, as well as Treg cells with increased suppressive capacity, can provide a favorable environment of successful transplant tolerance.^12^^,^^57^^,^^58^ Furthermore, in hematopoietic stem cell transplantation, high Treg cell:CD4 T cell ratios are associated with reduced acute graft-versus-host disease and reduced overall mortality.^58^ Importantly, *in vitro* expanded Treg cells with enhanced suppressive capacity have already entered clinical trials.^59^ Therefore, identifying genetic variants that regulate gene expression in a specific cellular context can inform development of more effective cell therapies. Our study provides an important advancement in mapping regulation of gene expression in Treg cells, and consequently, our results can benefit a range of clinical conditions.

### Limitations of the study

There are the following limitations to our study that should be considered. For example, a subset of loci where we were unable to link chromQTLs with eQTLs could also result from long-distance gene expression regulation, the eQTL being outside of our testing window, or from combinatorial subtle effects between multiple enhancers regulating the expression of individual genes. We also recognize instances of complex regulation of gene expression that will require targeted follow-up studies to fully uncover the functional role of disease variants. For example, we observed a complex pattern of colocalization between *CD28* eQTL, nearby actQTLs, and immune disease GWAS variants. The eQTL for *CD28*, the co-stimulatory receptor found on the surface of the majority of T cells, was specific to Treg cells and absent from naive T cells. The risk alleles for CEL and MS showed reversed effects on *CD28* expression and the acetylation of the peaks, implicating complex enhancer-mediated control of *CD28* expression under cell type and cell-state-specific mechanisms. Therefore, the results we describe here form the basis for hypothesis-driven functional follow-up studies into Treg-cell-mediated development of autoimmune and inflammatory diseases.

Finally, determining cell-type-specific QTL effects is challenging due to technical confounding factors between studies, including sequencing depth, different sample sizes across studies, different protocols of sample processing, etc. Although we performed numerous analyses to demonstrate our dataset captured true Treg-cell-specific effects, we recognize that some of the Treg-cell-specific effects that we identified here could be shared with other cell types. The dropping costs of single-cell transcriptomic technologies, higher gene capture efficiency, and increasing applicability to profile transcriptome of immune cells both in circulation and in tissues will map the cell-type- and context-specific gene expression regulation with high precision.

## STAR★Methods

### Key resources table

**Table:** 

REAGENT or RESOURCE	SOURCE	IDENTIFIER
**Antibodies**		

anti-CD4-APC, clone OKT4	BioLegend, San Diego, U.S.	Cat. no. 317416; RRID:AB_571945
anti-CD127-FITC, clone eBioRDR5	Thermo Fisher Scientific, Waltham, U. S.	Cat. no. 11-1278-42; RRID:AB_1907342
anti-CD25-PE, clone M-A251	BioLegend, San Diego, U.S.	Cat. no. 356104; RRID:AB_2561861
anti-FOXP3-BV421, clone 206D	BioLegend, San Diego, U.S.	Cat. no. 320123; RRID:AB_2561338
H3K4me3	Active Motif, Carlsbad, U.S.	Cat. no. 39915; RRID:AB_2687512
H3K27ac	Diagenode	Cat. no. C15410196; RRID:AB_2637079

**Biological samples**		

Lymphocyte cones were obtained with informed consent from healthy adults of Caucasian origin.	NHS Blood and Transplant, Cambridge and from the NHS Blood and Transplant, Oxford	REC 15/NW/0282, REC 15/NS/0060

**Chemicals, peptides, and recombinant proteins**		

TRIzol	Thermo Fisher Scientific	15596026
NEBNext® High-Fidelity 2X PCR Master Mix	New England Biolabs, Ipswich, U.S.	M0541L
Tn5 enzyme	Nextera	TDE1
EvaGreen dye	Biotium, Fremont, U.S.	#31000

**Critical commercial assays**		

EasySep® Human CD4^+^ T Cell Enrichment Kit	StemCell Technologies, Vancouver, Canada	Cat. no. 19052
iDeal ChIP-seq Kit for Histones	Diagenode, Liege, Belgium	C01010059
RNeasy Mini Kit	QIAgen, Hilden, Germany	74106
KAPA RNA HyperPrep Kit	Roche, Basel, Switzerland	KK8541
Nextera DNA Library Prep Kit	Illumina, U.S.	FC-131-1096
MinElute PCR Purification Kit	QIAgen, Hilden, Germany	28006
Nextera Index Kit	Illumina, U.S.	TG-131-2001

**Deposited data**		

Raw data generated in this study	EGA	https://wwwdev.ebi.ac.uk/ega/studies/EGAS00001003516
BLUEPRINT consortium CD4^+^ T cell and monocyte RNA-seq and ChIP-seq datasets	EGA	EGAD00001002671, EGAD00001002674, EGAD00001002673, EGAD00001002674
DICE project data	DICE project: Linking immune disease GWAS variants to genes and cell types, Date of approval: 2019-08-23	https://dice-database.org/
FANTOM5	Predefined enhancer-TSS bed sets	http://enhancer.binf.ku.dk/presets/enhancer_tss_associations.bed
Custom scripts and pipelines repository:	Treg_Multiomic	https://github.com/trynkaLab/

**Software and algorithms**		

GitHub (original codes supporting this work)	https://doi.org/10.5281/zenodo.6335757	https://github.com/TrynkaLab/Treg_Multiomics/tree/v1.0.1
BEAGLE 4.1	Browning et al.^60^	http://faculty.washington.edu/browning/beagle/beagle.html
VerifyBamID v1.0.0	Jun et al.^61^	https://github.com/statgen/verifyBamID/releases
STAR	Dobin et al.^62^	https://github.com/alexdobin/STAR/releases
subread package v1.5.1	Liao et al.^63^	http://subread.sourceforge.net/
skewer	Jiang et al.^64^	https://github.com/relipmoc/skewer
bwa	Li and Durbin.^65^	http://bio-bwa.sourceforge.net/
samtools	Li et al.^66^	http://samtools.sourceforge.net/
MACS2	Zhang et al.^67^	https://github.com/macs3-project/MACS
BEDTOOLS	Quinlan and Hall^68^	https://bedtools.readthedocs.io/en/latest/
QTLtools	Delaneau et al.^69^	https://qtltools.github.io/qtltools/
coloc v2.3–1	Giambartolomei et al.^31^	https://github.com/chr1swallace/coloc
TFmotifView	Leporcq et al.^70^	http://bardet.u-strasbg.fr/tfmotifview/
g:Profiler	Raudvere et al.^66^	https://biit.cs.ut.ee/gprofiler/gost
DESeq2_.1.14	Love et al.^71^	https://bioconductor.org/packages/release/bioc/html/DESeq2.html
ASEReadCounter (4.0.1.1)	Castel et al.^72^	https://gatk.broadinstitute.org/hc/en-us/articles/360037054312-ASEReadCounter

**Other**		

Lympholyte-H density gradient centrifugation.	(Cedarlane Labs, Burlington, Canada)	CL5020
Infinium® CoreExome-24 v1.1 BeadChip	Illumina	WG-331-1101

### Resource availability

#### Lead contact

Further information and requests for resources and reagents should be directed to and will be fulfilled by the lead contact, Gosia Trynka (gosia@sanger.ac.uk).

#### Materials availability

This study did not generate new unique reagents.

### Method details

#### Sample collection and Treg isolation

Lymphocyte cones were obtained with informed consent from donors at the NHS Blood and Transplant, Cambridge (REC 15/NW/0282) and from the NHS Blood and Transplant, Oxford (REC 15/NS/0060).

Leukodepletion cones were obtained from healthy adults of Caucasian origin. PBMCs were isolated using Lympholyte-H (Cedarlane Labs, Burlington, Canada) density gradient centrifugation. CD4^+^ T cells fraction of the PBMCs was obtained by negative selection using EasySep® Human CD4^+^ T Cell Enrichment Kit (Cat. no. 19052, StemCell Technologies, Vancouver, Canada), following the manufacturer’s instructions. Next, the CD4^+^ T cells were resuspended in the FACS staining buffer (2 mM EDTA and 0.5% FCS in PBS) at 10^8^ cells per mL. The cells were stained with the following antibody cocktail: anti-CD4-APC (30 μL/mL final volume, clone OKT4, Cat. no. 317416, BioLegend, San Diego, U.S.), anti-CD127-FITC and (30 μL/mL, clone eBioRDR5, Cat. no.11-1278-42, Thermo Fisher Scientific, Waltham, U. S.) and anti-CD25-PE (80 μL/mL, clone M-A251, Cat. no. 356104, BioLegend) for at least 30 min at RT in the darkness. The cells were washed copiously with FACS buffer and resuspended at 10^8^ cells per mL in full medium (IMDM, 10% FCS) and kept overnight at 4°C. Immediately before sorting, the cells were stained with DAPI, to discriminate between live and dead cells (Figure S1G). The CD4^+^, CD25^high^, CD127^neg^ population corresponding to Treg lymphocytes was sorted out for the downstream assays (Figures S1A and S1B). We sorted up to 3 million cells in order to carry out all of the downstream assays. In instances where this number was not reached we prioritised RNA-seq, followed by H3K27ac and H3K4me3 ChIP-seq, and finally ATAC-seq.

#### Sample summary

For all donors we were able to extract their sex based on their genotype and for 113 of the 124 donors we had access to their age (Figure S1D). The majority of the donors (78%) were genetically assigned males and were aged over 57 years of age (±11).

#### FACS staining

To verify the FOXP3 expression in the sorted Treg populations after sorting, the cells were stained for expression of CD4, CD25 and CD127 surface markers, and then stained with anti-FOXP3-BV421 antibody (5 μL/10^6^ cells, clone 206D, BioLegend) using the eBioscience™ Foxp3/Transcription Factor Staining Buffer Set (Thermo Fisher Scientific), according to the manufacturer’s instructions. We observed that the sorted cells were on average 80% FOXP3 positive (Figures S1B and S1E).

To define the proportions of memory and naive cells in the CD4^+^ population, an aliquot of 10^6^ cells after the CD4-enrichment were resuspended in 100 μL FACS buffer and stained with a cocktail of anti-CD4-APC and anti-CD127-FITC antibodies (3 μL each), anti-CD25-PE (8 μL) and anti-CD45RA-BV785 (4 μL, clone HI100, Cat. no. 304140, BioLegend), incubated at RT in the dark for at least 30 min, washed copiously with FACS buffer and analysed on BD Fortessa. The majority of the isolated Tregs were memory Tregs (median = 79%) (Figure S1F).

#### Culture and stimulation of isolated Tregs

Whole blood samples were obtained from ten healthy adults, aged from 22 to 39 years. Live regulatory T cells (CD4^+^ CD25high CD127low) were isolated as described in Sample collection and Treg isolation. Cells were grown in Iscove’s Modified Dulbecco’s Media (IMDM) (Life Technologies, Paisley, UK), supplemented with 10% human serum (HS), 50 U/mL penicillin and streptomycin (Life Technologies) and 100 U/mL recombinant human IL-2 and incubated at 37°C in a humidified atmosphere of 5% CO_2_. Cells were activated using PMA (5-10 ng/μL) with ionomycin (200 ng/μL) (Sigma-Aldrich) overnight (18 hours).

#### SNP genotyping and imputation

A total of 551,839 genetic markers were genotyped using the Infinium® CoreExome-24 v1.1 BeadChip by Illumina. After SNP QC (MAF >10%, SNP call rate >95%, Hardy-Weinberg equilibrium (HWE) p value < 0.001) we retained 243,820 variants in our dataset. Samples with call rate <95% were removed from the analysis. After quality control per individual, the total genotyping call rate reached >99%. We performed imputation using BEAGLE 4.1 with a reference panel comprising the 1000 Genomes Phase 3^60^ and the UK10K^73^ samples (modelscale parameter = 2). Following imputation we required allelic R-squared (AR2) ≥ 0.8, HWE p value < 0.001, and MAF >5% in both the analysed cohort and in the reference panel. We excluded 1,934 multiallelic polymorphisms from further analysis which resulted in 5,761,739 variants in our final dataset. Of those, 617,318 were insertion-deletions (INDELs). All genetic variant coordinates were lifted over to GRCh38.

Our samples clustered with the European populations included in the 1000 Genomes project (Figure S2F). We removed 1 sample due to high relatedness (identity by state, pi_hat >0.2). We used VerifyBamID v1.0.0^61^ with the genotype information along with all the functional genomics sequencing assays (see below) to verify no sample swaps were present in the final dataset.

#### RNA-seq

For RNA-seq experiments, 0.5 × 10^6^ sorted Treg cells were washed with ice-cold PBS and resuspended in TRIzol (Thermo Fisher Scientific). After a standard phenol/chloroform isolation step, the total RNA contained in the upper, aqueous phase was further purified with RNeasy Mini Kit (QIAgen, Hilden, Germany), according to the manufacturer’s instructions. The RNA libraries were constructed using KAPA RNA Hyper-Prep Kit (Roche, Basel, Switzerland), following a standard automated protocol. The libraries were multiplexed and sequenced at 75 bp PE on an Illumina HiSeq V4 to yield on average 57 million reads per sample.

#### ATAC-seq

ATAC-seq was performed according to protocol,^74^ with the following modifications. After sorting, the T cells were washed with ice-cold PBS and resuspended in sucrose buffer (10 mM Tris pH 8, 3 mM CaCl2, 2 mM MgOAc, 1 mM DTT, 0.32 M sucrose, 0.5 mM EDTA, 0.25% TritonX-100), followed by 5 min incubation on ice to isolate the nuclei. Isolated nuclei were washed once with 1x TD buffer (Tagment DNA Buffer, Nextera DNA Library Prep Kit, Illumina, U.S) and resuspended in 50 μL 1x TD buffer containing 2.5 μL of Tn5 enzyme (TDE1, Nextera). The reaction was carried out at 37°C, mixing and then stopped by addition of 250 μL of buffer PB (MinElute PCR Purification Kit, QIAgen, Hilden, Germany). The DNA was then purified on MinElute columns according to the manufacturer’s instructions and eluted in 10 μL sterile ddH_2_O. The libraries were amplified using the NPM mix (Nextera PCR Master Mix from Nextera DNA Library Prep Kit) and Index adapters i7 and i5 (Nextera Index Kit, Illumina, U.S), according to the manufacturer’s instructions. The number of amplification PCR cycles for each sample was determined individually by performing a qPRC reaction of 7.5 μL aliquote of the mix with an addition of the EvaGreen dye (Biotium, Fremont, U.S.). The amplified libraries were SPRI purified (upper cut 0.5x, lower cut 1.8 x) on a Zephyr G3 SPE Workstation (PerkinElmer, Waltham, U.S.), multiplexed and sequenced at 75 bp PE on an Illumina HiSeq V4 to yield on average 112 million reads per sample.

#### H3K4me3 and H3K27ac ChIPmentation-seq

The ChIPmentation-seq (ChM-seq) protocol was performed on 100,000 sonicated cells according to the protocol presented in Schmidl et al.^75^ and adapted to work with the iDeal ChIP-seq Kit for Histones (Diagenode, Liege, Belgium).

After sorting, the cells were resuspended in pre-warmed full medium (IMDM, 10% FCS) at 1-2 million cells per mL and allowed to recover in the incubator (37°C, 5% CO_2_) for at least 30 min. The cells were then fixed by addition of formaldehyde to medium to a final concentration of 1% and 5 min incubation at 37°C, followed by quenching with glycine for 5 min at a final concentration of 125 mM min at RT with mixing. The cross-linked cells were subsequently washed twice with ice-cold PBS and snap-frozen by immersion in liquid nitrogen.

0.5 × 10^6^ frozen cells were resuspended in 250 μL buffer iL1 with proteinase inhibitors cocktail (iDeal ChIP-seq Kit for Histones, Diagenode) and incubated for 10 min at 4°C on the Bohemian wheel. The samples were then spun down, and resuspended first in buffer iL2 with proteinase inhibitors, then in iS1 with proteinase inhibitors, in both cases also for 10 min at 4°C. The cells were then sonicated in buffer iS1 using the Bioruptor® Pico sonication device (Diagenode) to achieve fragment sizes distribution below 3 kb.

Sonicated chromatin from 100,000 cells was used for an overnight immunoprecipitation reaction with 1 μg of antibody, either against H3K4me3 (Catalog No: 39915, Active Motif, Carlsbad, U.S.) or H3K27ac (Cat. no. C15410196, Diagenode).

The samples in deep-well plates were then washed twice for two minutes with 150 μL of each of the buffers: iW1, iW2, iW3 (iDeal ChIP-seq Kit for Histones, Diagenode) and then with 10 mM Tris pH 8. All the washes in this protocol were performed using an Agilent Bravo Automated Liquid Handling Platform (Agilent, Santa Clara, U.S.). After the second Tris wash, a ChIPmentation reaction on the beads was conducted following the protocol outlined in Schmidl et al. Briefly, a mix containing 1 μL Tn5 from the Nextera kit was added to the beads and incubated for 10 minutes with vigorous mixing at 37°C. Next, the reaction mix was removed using Bravo, and additional washes were performed, two with buffer iW3, followed by two washes with buffer iW4. The enriched DNA was eluted from the beads by incubation with 67 μL buffer iE1 (1 h, RT, vigorous shaking). 3 μL of iE2 buffer were then added to each sample and the cross-linking was reversed by an overnight incubation at 65°C in a thermocycler.

The DNA was then purified twice using SPRI beads at 1.6x ratio using a Zephyr G3 SPE Workstation. The libraries were amplified following the ATAC-seq library amplification protocol, but using NEBNext® High-Fidelity 2X PCR Master Mix (New England Biolabs, Ipswich, U.S.). Finally, the ChIPmentation libraries were sequenced to a depth of at least 13 million reads per sample and an average of 75 million reads per sample.

### Quantification and statistical analysis

#### RNA-seq data processing

Reads were aligned to the GRCh38 human reference genome using STAR^62^ and the Ensembl reference transcriptome (version 87). Gene counts were performed using featureCounts tools from the subread package v1.5.1^63^ and only assigned reads were used for further processing (59.26% of reads were assigned; Figure S2D). We excluded short RNAs and pseudogenes from the analysis. We quantile normalised the gene expression values and corrected for GC-content using the CQN method.^76^ We kept 12,059 genes with average count per gene across all donors greater than 25.

#### Chromatin marks data processing

Reads were trimmed using skewer^64^ and aligned to the GrCh38 assembly of the human genome using bwa^65^ and employing the mem algorithm. Multi-mapping and duplicated reads were removed using samtools.^66^ For ATAC-seq data, reads aligning to the mitochondrial chromosome were also removed. Only reads mapping to autosomes were maintained. A median of 30, 27 and 40 million reads in the ATAC-seq, H3K4me3 and H3K27ac passed this QC, respectively.

Peak calling was performed using MACS2^67^ independently on each donor for quality control purposes. For ATAC-seq peaks were called using the standard MACS2 model and specifying --nomodel --shift −25 --extsize 50 on fragment BED files (this is, both reads of a pair were merged into a single fragment). We generated a combined treatment set per histone mark by merging an equal number of reads per donor to reach the combined merged input size of 223 million reads. H3K4me3 peaks were called using the standard narrow peak MACS2 model, specifying -f BAMPE --keep-dup all, then we selected only the peaks with q-value < 0.01 and fold-change greater than 2. H3K27ac broad peaks were called using the standard broad peaks macs2 model, specifying -f BAMPE --broad --nomodel --extsize 146 --keep-dup all, then we selected only the peaks with q-value < 0.001 and fold-change greater than 2.

Samples with less than 10,000 peaks (median: ATAC 36,331, H3K4me3 22,815, H3K27ac 68,626), fraction of reads in peaks (FRiP) lower than 10% (median: ATAC 23%, H3K4me3 52.64%, H3K27ac 63.9%) (Figure S2D), or, for ATAC-seq, an abnormal insert profile (defined as a ratio of short inserts (<150 bp) over long inserts (>150) smaller than 1.5; average 2.03) were discarded. Additionally, the samples that did not cluster with the corresponding group in principal component analysis (considering log2 transformed number of reads in genomic bins of 10,000 bp, after normalization by library length) were discarded from further analysis. Finally, a total of 73 (62%), 88 (79%) and 91 (78%) individuals passed these filters for ATAC, H3K4me3 and H3K27ac samples, respectively. Sixty-two donors passed QC steps for all the tested genomic assays (RNA, ATAC, H3K4me3 and H3K27ac).

In order to define a consensus set of peaks per chromatin assay, we performed a merged peak calling combining reads from all the donors. We downsampled each donor sample using samtools to 2 million fragments per ATAC-seq assay, 1.87 million read pairs per H3K4me3 and 1.86 million read pairs per H3K27ac assay in order to reach similar read counts to the sequenced inputs. We used the MACS2 parameters described above and specified --keep-dup all. Then, to ensure a sufficient number of reads per peak, only ATAC-seq peaks with at least 10 reads in 80% of the samples, and ChM-seq peaks with fold enrichment ≥ 2 and adjusted p value < 0.001, were maintained in the final set. The consensus sets were 39,642 ATAC-seq narrow peaks, 40,285 H3K4me3 ChM-seq narrow peaks and 34,457 H3K27ac broad peaks. The peak overlap between the assays as calculated using bedtools intersect, the distance to the closest transcription start site (TSS) is shown in Figures S2A and S2B). The median length was 523 bp, 794 bp and 4501.5 bp for the ATAC-seq peaks, H3K4me3 peaks and H3K27ac peaks, respectively. The median number of read pairs in each peak (calculated using featureCounts -p -C -D 5000 -d 50) per sample amounted 37.95 in ATAC-seq, 16.34 in H3K4me3 ChM-seq and 123.38 in H3K27ac ChM-seq (Figure S2C).

We assayed 17, 8, 15 and 15 samples twice (same donors were recruited at two different time points) for RNA, ATAC-seq, H3K27ac ChM-seq and H3K4me3 ChM-seq, respectively. We observed high correlation between both technical (same donors different times) and biological replicates (different donors) (R2 > 0.8) in all assays. We observed greater correlation between technical than between biological replicates, as expected (Figure 2E).

In order to compare QTL effects between the Tregs and the naive CD4 T cells and monocytes in the BLUEPRINT dataset, we combined the H3K27ac broad peaks called independently in each cell type into a consensus set of peaks. Overlapping peaks were merged using the merge option implemented in bedtools.^68^

Genome browser data was constructed using the MACS2 -B flag and reads were normalised to signal per million. The fold-enrichment was calculated using the input background and finally bigwigs were constructed using bedGraphToBigWig command from the UCSC suite of tools.^77^ Coverage plots were generated using an adapted version of the wiggleplotr R Bioconductor package.^78^

#### Quantitative trait locus mapping (QTLs)

Prior to the QTL analysis we removed genes and peaks mapping to the MHC region (chr6: 20,000,000-40,000,000) and only kept the autosomal chromosomes. We used linear regression implemented in the QTLtools^69^ software to map cis QTLs. For the gene expression we used a 500 kbp cis-window around the gene, while for the three chromatin mark assays we used a 100 kbp cis-window around the defined peak. In chromQTL mapping we were directly assaying the chromatin features and we were focusing specifically on those loci with chromQTL SNPs located in the controlled peaks, therefore we reasoned that a smaller window was more appropriate.^79^ As covariates we used the top 13, 30, 22 and 33 principal components that each explained up to 1% of the observed variance in the RNA, ATAC, H3K27ac and H3K4me3, respectively. We used the “--permute 10000” to obtain permutation p-values for the top most significantly associated variant for each gene or peak. We then used eigenMT^80^ to correct for the number of genes or peaks tested and used a cut-off of 5% FDR, as determined by power analysis (Figure S7).

To perform comparative analysis between Treg and monocytes and naive T cell eQTLs and actQTLs we downloaded the RNA-seq and ChIP-seq datasets generated by the BLUEPRINT consortium^24^ from EGA (EGA: EGAD00001002671, EGAD00001002674, EGAD00001002673, EGAD00001002674) and processed the data using the same workflow as described above. We included the top 16 and 14 PCs for these datasets in the monocytes and naive T cells eQTL analyses respectively. We included the top 13 and 17 PCs for these datasets in the monocytes and naive T cells actQTL analyses, respectively. We chose this dataset because Tregs and naive T cells are closely related cells of adaptive immunity, while monocytes fall into a more distant cell type of the innate immune arm. Furthermore, the datasets are of similar size (197 and 169 individuals) to the Treg dataset and all the individuals were of British origin.

We used the following three criteria to define an eQTL and actQTL as cell type specific when comparing monocytes, naive and regulatory T cells: (i) the gene was expressed in one cell type only or the peak was only present in one cell type, (ii) the gene or peak was a significant QTL in one cell type (FDR ≤0.05) and not in the other (FDR >0.2) and (iii) if the same gene or peak was a QTL in both cell types and the LD between the top QTL variant in regulatory T cells and any of the significant associated signals in naive T cells was lower than R^2^ < 0.2.

#### Allele-specific expression analysis

We used ASEReadCounter^72^ from the Genome Analysis ToolKit (GATK) to count the number of allele-specific fragments overlapping each variant in the RNA-seq data. We used ‘-U ALLOW_N_CIGAR_READS -dt NONE --minMappingQuality 10 -rf MateSameStrand’. We filtered out variants covered by less than 8 reads, as well as variants that fell within regions with low mappability or that displayed mapping bias using simulated data, as outlined in Castel et al.^72^ annotated each variant to its overlapping gene. Significant allele-specific events were calculated using a binomial test, where the null was defined by taking the average reference ratio across all heterozygous sites for each sample.

#### Colocalization of QTL signals with immune disease GWAS

We used *coloc* v2.3–1^31^ with the default priors to test for colocalization between molecular QTLs and GWAS SNPs listed in Tables S2 and S3. We included in our analysis the summary stats for the genome-wide association studies of 14 immune related diseases: allergic diseases (ALL),^39^ ankylosing spondylitis (AS),^81^ asthma (AST),^82^ celiac disease (CEL),^83^ multiple sclerosis (MS),^84^ primary biliary cirrhosis (PBC),^85^ psoriasis (PS),^86^ rheumatoid arthritis (RA),^87^ systemic lupus erythematosus (SLE),^88^ type 1 diabetes (T1D),^89^ vitiligo (VIT),^90^ inflammatory bowel disease (IBD), Crohn’s disease (CD) and ulcerative colitis (UC).^91^ We selected these diseases because they had more than 40 GWAS associated independent loci at p-value < 10^−5^. As controls we tested two non-immunological traits with a similar number of loci, type-2 diabetes (T2D)^92^ and depression (DEP).^93^ CD, UC and IBD were counted as a single disease when counting for number of colocalizing diseases per gene or peak. Similarly for ALL and AST. *Coloc* tests five hypotheses for colocalization. Hypothesis zero (PP.H0) tests whether there is any association at all, PP.H1 and PP.H2 test whether there is an association with just one or the other study, PP.H3 tests whether the signal from GWAS and QTL is due to two independent SNPs, and PP.H4, test if the association between GWAS and QTL is due to a shared causal variant.

Prior to colocalization, we repeated the QTL mapping in chromatin features using a 500 kbp window, to run *coloc* at a larger window. We ran *coloc* on a 400-kb region centered on each lead eQTL and chromQTL variant that was less than 100 kb away from a GWAS variant (nominal p value < 10^−5^). We only kept the colocalizations between QTLs and non-HLA GWAS loci if there were more than 50 SNPs tested. To claim a true colocalizing signal we required PP.H4 to be equal or greater than 0.8^3^. In order to decrease the number of false positive findings in our Treg dataset, we focused on the colocalization results with common immune disease variants (MAF >10%). Colocalizations between Treg QTLs and disease GWAS signals were the lead QTL variants and the lead GWAS variant had R^2^ LD < 0.5 were discarded. For GWAS loci with 10^−5^ > p value > 5 × 10^−8^, and colocalizing with Treg QTLs we verified in the original publications that there was a replication cohort and the final GWAS p value was lower than genome wide significance.

#### Gene expression variance deconvolution

To estimate the contribution of the genetic component and chromatin marks to the transcriptome variance we fitted a multivariate linear model to the expression of each gene. Therefore, the dependent variable in the model was the gene expression, while the independent variables were the genetic variants and/or chromatin mark. We regressed out the PCs included in the analysis of each chromatin mark previous to model calculation. As described in de Bakker et al.,^94^ we performed an initial variable selection step by identifying the genetic variants or chromatin features in a +/−150 kb window that significantly correlated with gene expression (Spearman p value < 0.05). In order to keep only independent variables in the set of predictors, in the instances where pairs of genetic variants or chromatin features were correlated (Spearman correlation >0.4), we removed the variable with a lower correlation with gene expression. To determine the total variance explained we used the adjusted R^2^ of the model where we included all the independent variables from genetic variants and chromatin features. The individual contribution of genetic variants or each chromatin mark, or combination of the genetic variants and chromatin marks, was obtained by subtracting the R^2^ estimates of the models that excluded genetic variants or individual chromatin marks, or their combinations, from the R^2^ of the model with the total variance explained by the combination of genetic variants and all chromatin marks.

#### SNP functional annotation

All the lead variants (and their proxies, R^2^ > 0.8) of every significant caQTLs, actQTLs, and promQTLs were annotated if a variant was overlapping an ATAC, H3K27ac or H3K4me3 peak (Figure 1D shows the number of polymorphisms included in the different categories, categories are mutually exclusive and a variants is assigned to the category with most functional support).

#### LD loci definition and classification

A LD locus comprises a ±150 kb window around the region defined by the lead and proxy variants (R^2^ > 0.8) for each GWAS signal that colocalizes with a Treg QTL. Loci with significant colocalization signals were classified as: i) Tier 1, at least one colocalization with an eQTL and at least one colocalization with a chromQTL; ii) Tier 2, at least one colocalization with an eQTL, no colocalization with chromQTL; and iii) Tier 3, at least one colocalization with a chromQTL (Figure 3; Tables S2 and S3). Loci in Tier 1 and Tier 3 in which variants overlapped with the regulated (i.e. colocalizing) chromatin QTL peak were prioritized as functional.

#### Known drug tractability evidence analysis

We used the Open Targets Platform to extract information on drugs that support target-disease associations provided by ChEMBL.^41^ We retrieved the ‘known_drug’ evidence for all genes via the Open Targets API using the python client (Open Targets data release February 2020). When gathering data from OpenTargets we summarized the extracted data based on the website’s recommendations (https://platform-docs.opentargets.org/target/tractability).

#### Differential gene expression analysis

RNA-seq reads were obtained and processed as described in RNA-seq and in RNA-seq data processing. Genes with at least 25 copies in at least three samples were kept, for a final table of 16,645 genes. Differential expression analysis was performed using DESeq2_.1.14^71^ Wald test, setting alpha at 0.05 and lfc at 1. Differentially expressed genes that mapped within a +/− 150 kb window from a chromQTL in Tier 3 loci were annotated as candidate stimulation specific eQTL genes.

#### FANTOM5 CAGE data integration

The CAGE data in FAMTOM5^35^ provides predefined human enhancer-TSS pair sets (note that the use of one enhancer can be correlated with the TSS expression levels of several genes and vice-versa). Using “bedtools intersect” we defined the enhancers that overlapped Treg colocalizing chromQTL peaks. Then, we linked the Treg chromQTLs to the corresponding TSS.

#### Transcription factor binding site (TFBS) enrichment analysis

We used TFmotifView,^70^ which enables the user to input a set of chromosomal regions and perform a TFBS enrichment analysis on all TFs included in the JASPAR2020 database. This tool uses a custom set of regions as background. We used all Treg colocalizing actQTLs and compared them to actQTLs shared with naive CD4^+^ T cells. We followed-up on the set of enriched TFs by using g:Profiler,^95^ to identify enriched pathways.

## Data Availability

• All raw data produced here and existing data from multiple sources have the accession numbers listed in the key resources table. Access to DICE project data was authorized via dbGaP to Dr. Gosia Trynka (Project: Linking immune disease GWAS variants to genes and cell types, Date of approval: 2019-08-23). We used FANTOM5 predefined enhancer-TSS bed sets. The gene expression dataset is integrated into the eQTL catalogue (https://www.ebi.ac.uk/eqtl/). All colocalization results can be browsed via this website: https://www.sanger.ac.uk/science/tools/treg-colocalisation/treg-colocalisation/ • All original code has been deposited at Zenodo and is publicly available as of the date of publication. DOIs are listed in the key resources table. • Any additional information required to reanalyze the data reported in this paper is available from the lead contact upon request.

## Supplemental information

Document S1. Figures S1–S7

Table S1. Overview of the genomic assays performed across the donors: Sample collection and Treg cell isolation, related to STAR Methods

Table S2. Treg cells significant QTL summary stats after eigenMT correction, along with cell type sharing information, related to Figure 2 Genetic variants are named using the following convention: Chromosome, base pair, reference allele, alternative allele, separated by “_”, e.g. chr17_39895095_A_G. Peaks are named using the following convention: Chromosome, start base pair, end base pair, type of a chromatin mark, separated by “_”, e.g. chr17_39924388_39928854_H3K4me3. All coordinates are in the GRCh38 build

Table S3. Colocalizing GWAS loci, related to Figure 3 Column legend: *Tier*: functional classification of each GWAS locus (Tier 1: eQTL+ chromatin QTL; Tier 2: eQTL only; Tier 3: chromQTL only) *LD locus*: defined as the region containing all SNPs in R^2^ ≥ 0.8 with the reported lead GWAS SNP and extended by 50kb to each side. Overlapping loci were merged. *Disease colocalization*: disease which is colocalizing with the QTL signal *QTLs*: type of colocalizing QTLs *Features*: coordinates of the peaks of colocalizing chromatin marks for chromQTLs and/or gene symbol for eQTLs *GWAS lead SNP (GRCh37):* GWAS lead variant position (build GRCh37 nomenclature) *GWAS lead SNP (GRCh38):* GWAS lead variant position (build GRCh38 nomenclature) *GWAS allele*: allele of the GWAS SNP *GWAS effect*: direction of the effect of the GWAS allele *QTL SNPs (GRCh37):* Lead variants of the colocalizing QTLs (build GRCh37 nomenclature) QTL *SNPs (GRCh38):* Lead variants of the colocalizing QTLs (build GRCh38 nomenclature) *R2*: LD between the GWAS lead SNP and the QTL lead SNPs *GWAS SNP pval*: p-value of the GWAS lead SNPs in the disease GWAS summary stats *GWAS SNP QTL pval*: p-value of the GWAS lead SNPs in the QTL summary stats *GWAS SNP QTL slope*: the slope of the GWAS lead SNP in the QTL effect summary stats *QTL SNPs pval:* p-value of the lead QTL variant *PP.H4:* Colocalization values output from coloc

Table S4. Functionally refined colocalizing GWAS loci: SNP functional annotation, related to STAR Methods Column legend: *LD locus SNPs*: Number of genetic variants in the LD locus (R^2^ ≥ 0.8 with GWAS lead) *SNPs in chromatin QTL*: Number of LD locus SNPs mapping within the colocalizing chromatin QTL *SNPs in ATAC Peak*: Number of SNPs overlapping chromatin QTL and in ATAC-seq peaks

Table S5. Candidate gene nomination in chromQTLs: Differential gene expression analysis and FANTOM5 CAGE date integration, related to STAR Methods *Column legend*: *GWAS lead SNPs*: colocalizing lead GWAS SNP (build GRCh37 nomenclature) *GWAS lead SNP bp (GRCh38)*: colocalizing lead GWAS SNP build GRCh38 nomenclature *DEG*: differentially expressed genes in stimulated Tregs *ASE:* differentially expressed genes in stimulated Tregs that display allele specific expression with regards to the GWAS variant (binomial test FDR < 0.05) *Enhancer-TSS*: Transcription start sites linked to colocalizing chromatin QTL using CAGE data from FANTOM5

Table S6. Transcription factor motif enrichment in Treg cell actQTLs versus Treg cell actQTLs shared with naive T cells: Transcription factor binding site (TFBS) enrichment analysis, related to STAR Methods

Table S7. Linkage disequilibrium and functional annotation information for all the SNPs in each LD locus: LD loci definition and classification, related to STAR Methods *Column legend*: *SNP GRCh38*: SNP in build GRCh38 nomenclature *SNP GRCh37*: SNP in build GRCh37 nomenclature *LOCUS*: LD locus in which the SNP is included *LEAD GRCh38*: GWAS lead variant in build GRCh38 nomenclature *LEAD GRCh37*: GWAS lead variant in build GRCh38 nomenclature *chromQTL*: chromatin QTL peak overlapped by the SNP (chr:start-end, build GRCh38 coordinates) *Extra ATAC Peak*: ATAC-seq peak overlapped by the SNP (chr:start-end, build GRCh38 coordinates)

Table S8. Small molecule and antibody tractability clues for the colocalizing gene targets and genes linked to chromQTLs, related to Figure 5 Genes that had no evidence for both small molecules and antibodies were omitted.

Table S9. Treg cell eQTLs colocalizing with immune diseases that are targeted by drugs currently in clinical trials, related to Figure 5

Table S10. Treg cell eQTLs colocalizing with immune diseases that have an immune disease phenotype in mouse models, related to Figure 5 Loss of function (LoF) and gain of function (GoF) are broadly used here for anything that leads to severely decreased or increased expression, accordingly.

Document S2. Article plus supplemental information
