# Supplementary material for: Immune disease variants modulate gene expression in regulatory CD4+ T cells
Source: Cell Genom. 2022 Apr 6;2(4):100117. doi: 10.1016/j.xgen.2022.100117 (PMC9010307; doi:10.1016/j.xgen.2022.100117)
Supplement: Document S1. Figures S1–S7 [file mmc1.pdf]

Cell Genomics, Volume *2*

Supplemental information

Immune disease variants modulate gene expression in regulatory CD4$^+$ T cells

Lara Bossini-Castillo, Dafni A. Glinos, Natalia Kunowska, Gosia Golda, Abigail A. Lamikanra, Michaela Spitzer, Blagoje Soskic, Eddie Cano-Gamez, Deborah J. Smyth, Claire Cattermole, Kaur Alasoo, Alice Mann, Kousik Kundu, Anna Lorenc, Nicole Soranzo, Ian Dunham, David J. Roberts, and Gosia Trynka

Supplementary Figures

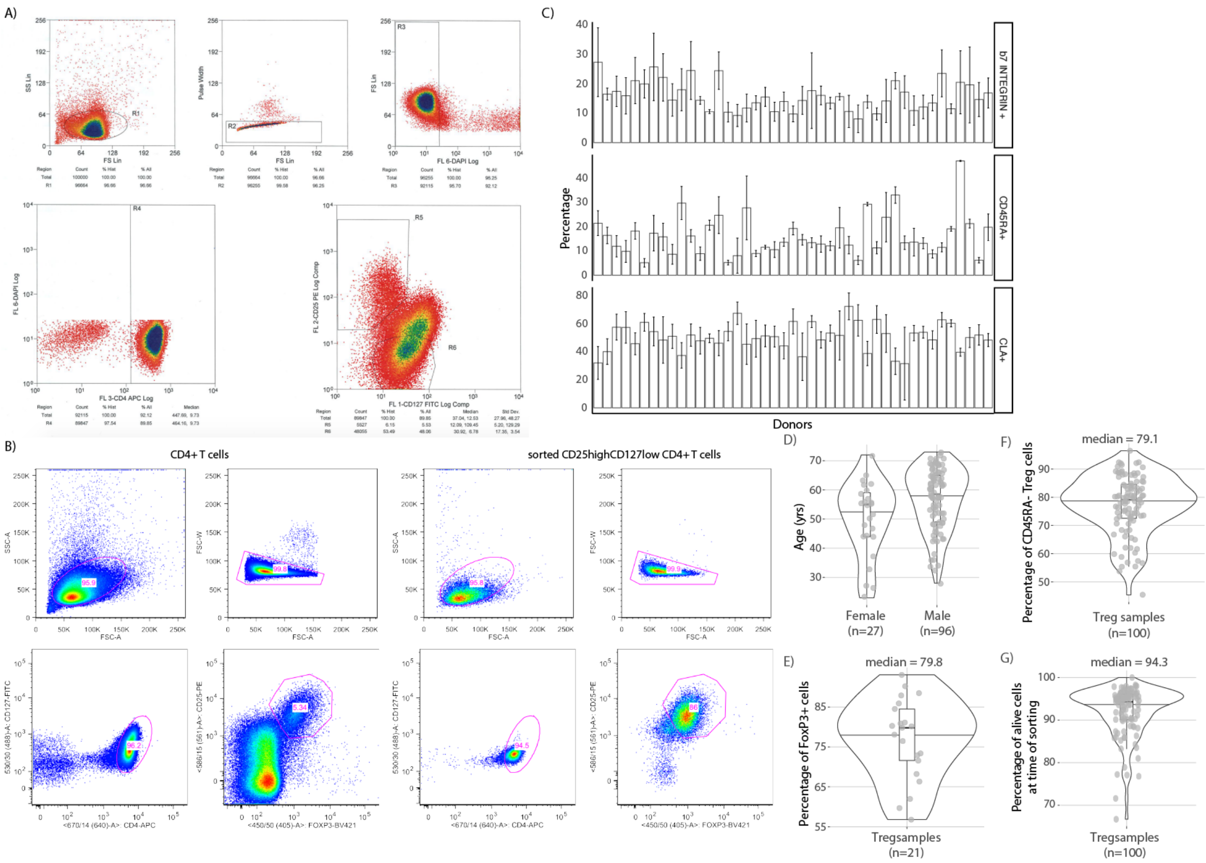

Supplementary Figure 1. Gating strategy for Treg isolation and validation. A) Gating used during the Treg cell sorting step. **B)** Gating strategy for the validation of Treg identity via FoxP3 staining. **C)** Percentage of regulatory T cells expressing b7 integrin, CD45RA and CLA directly after a blood draw. The same donors were assayed up to 3 times across multiple months. **D)** Donor age and sex distribution. **E)** Percentage of FOXP3+ cells per sample. **F)** Percentage of CD45RA- cells per sample. **G)** Percentage of alive cells as determined by DAPI at time of sorting. Related to STAR Methods: FACS staining

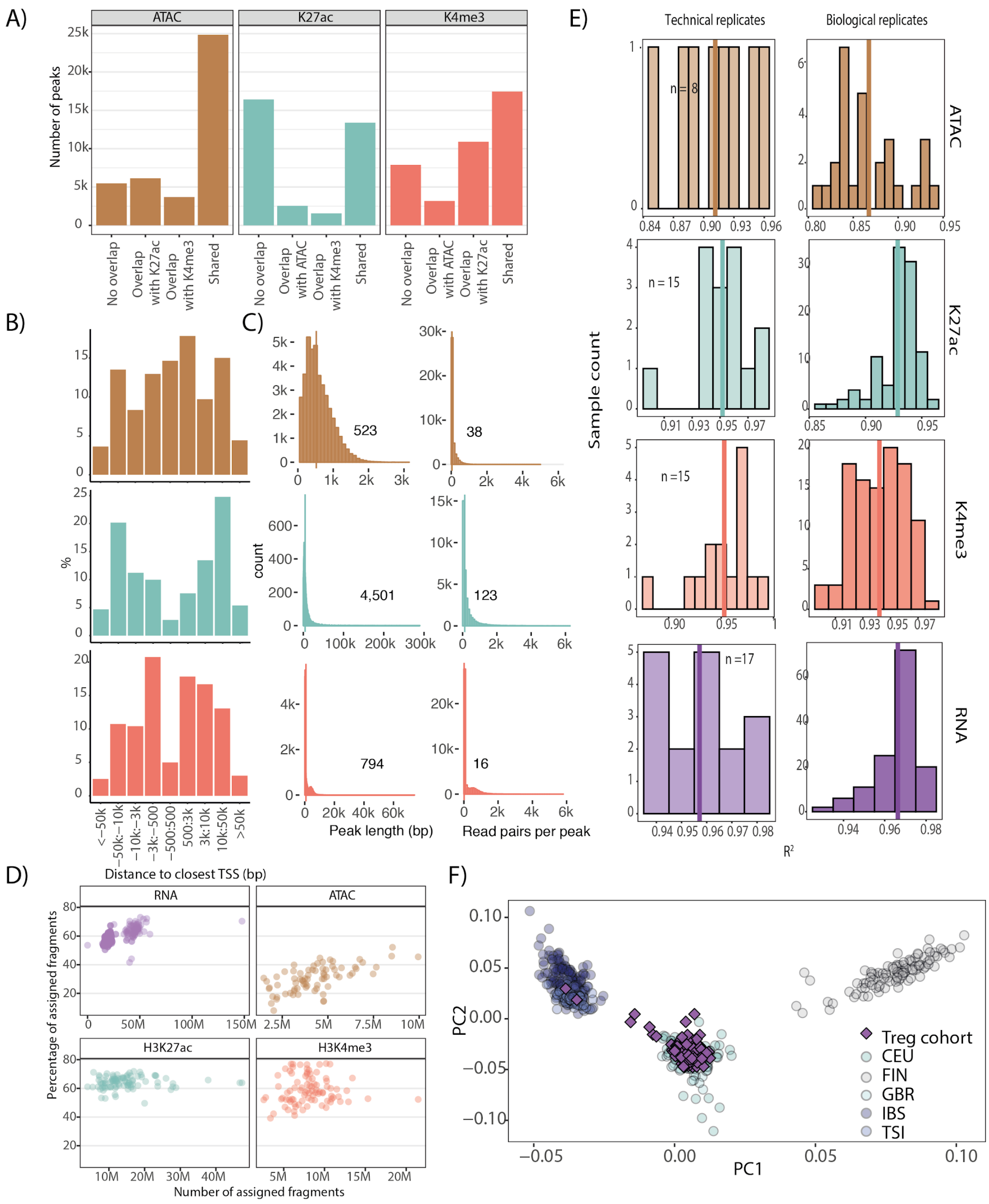

Supplementary Figure 2. Summary statistics. A) Peak numbers and peak overlaps across chromatin assays. In different colours are the numbers of peak overlaps per chromatin mark. **B)** Distribution of chromatin features in the proximity of the closest TSS. **C)** Distribution of peak length (left) and number of read pairs per peak (right). Median values are shown. **D)** Relationship between the total number of fragments per assay and the percentage of assigned fragments (sequenced read pairs) **E)** Correlation between technical and biological replicates in the different assays. **F)** Projection of the genotyped samples from our Treg study onto the European cohorts included in the 1000 Genomes Project. CEU: Utah Residents (CEPH) with Northern and Western European Ancestry; FIN: Finnish in Finland; GBR: British in

England and Scotland; IBS: Iberian Population in Spain; TSI: Toscani in Italia. Related to STAR Methods: SNP genotyping and imputation

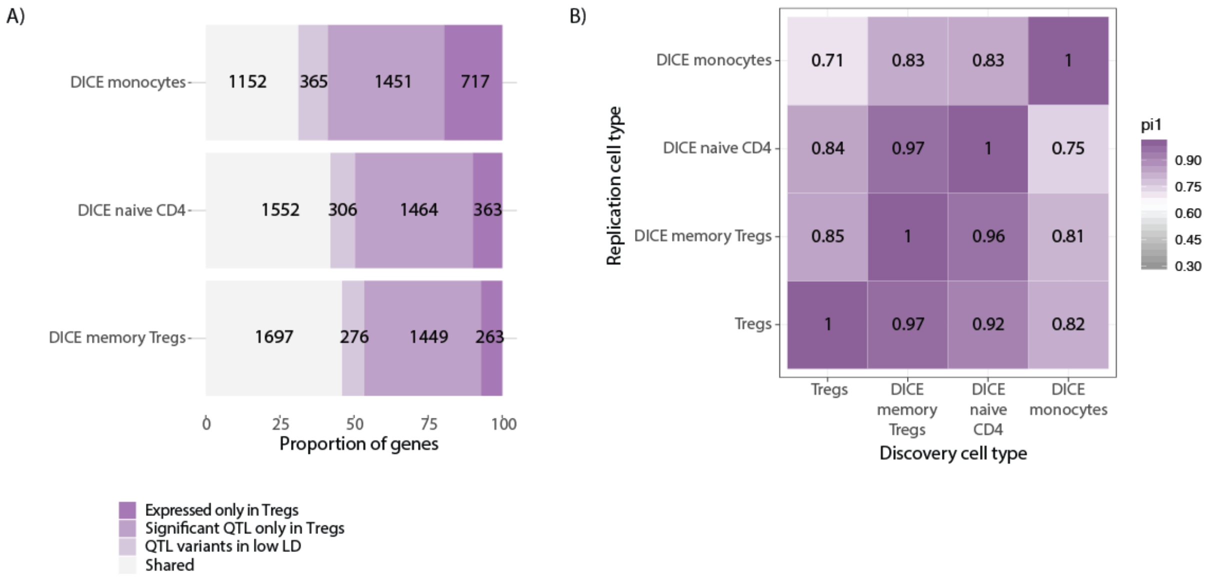

Supplementary Figure 3. Treg eQTLs comparisons with immune cell types assayed in the DICE consortium. A) Classification of Treg eQTLs specific to Tregs in relation to different immune cell types assayed in DICE. **B)** Pi1 scores between pairwise samples selected from DICE, Blueprint and the Treg eQTL dataset produced here. Related to Figure 2.

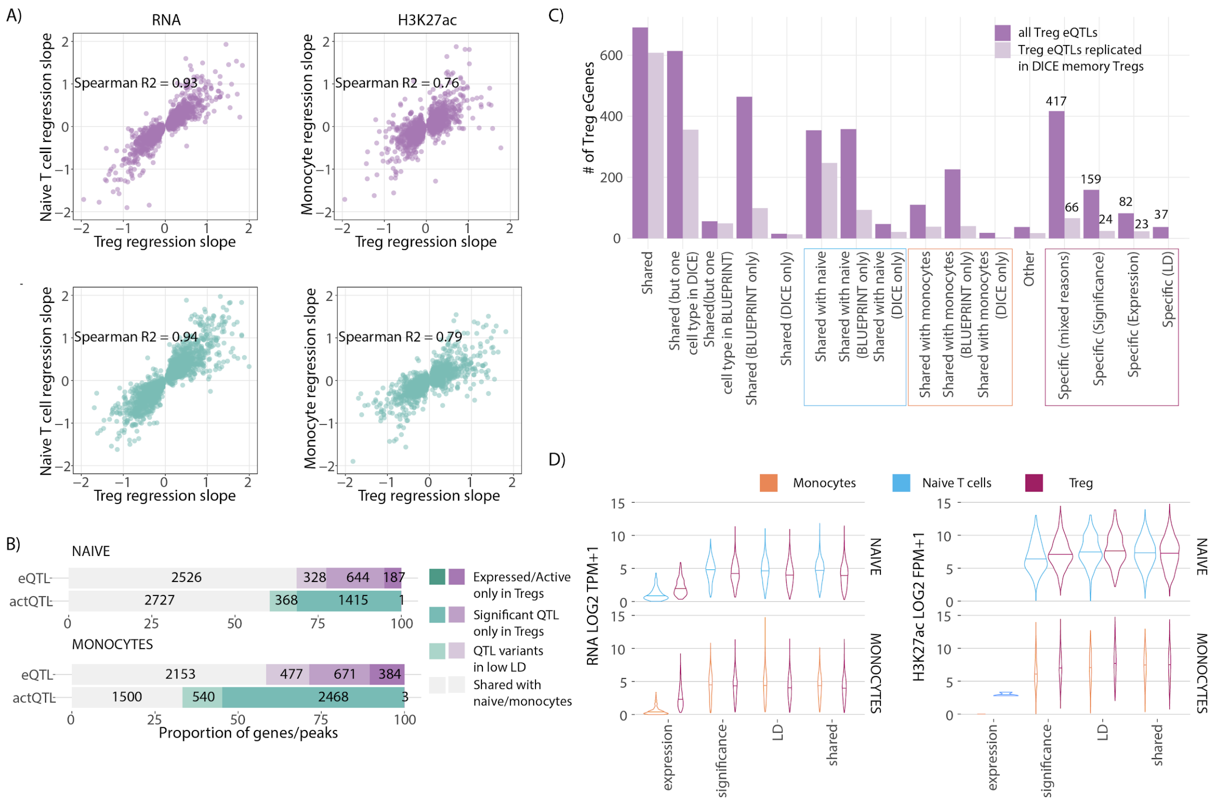

Supplementary Figure 4. Treg eQTLs and actQTLs comparisons with CD4+ naive cells and monocytes assayed in the BLUEPRINT. A) Correlation between the regression slopes for the top eQTL and actQTL variants discovered in CD4+ naive and regulatory T cells. Regression slope of the top eQTL and actQTL variant in regulatory T cells plotted against the slope for the same variant-gene pair in naive T cells. **B)** Classification of Treg eQTLs and actQTLs specific to Tregs in relation to naive T cells and monocytes. **C)** Classification of Treg eQTLs and actQTLs specific to Tregs in relation to naive T cells and monocytes from both the BLUEPRINT and the DICE consortia. **D)** Levels of gene expression and peak height across the QTL classifications in the different cell types. Related to Figure 2.

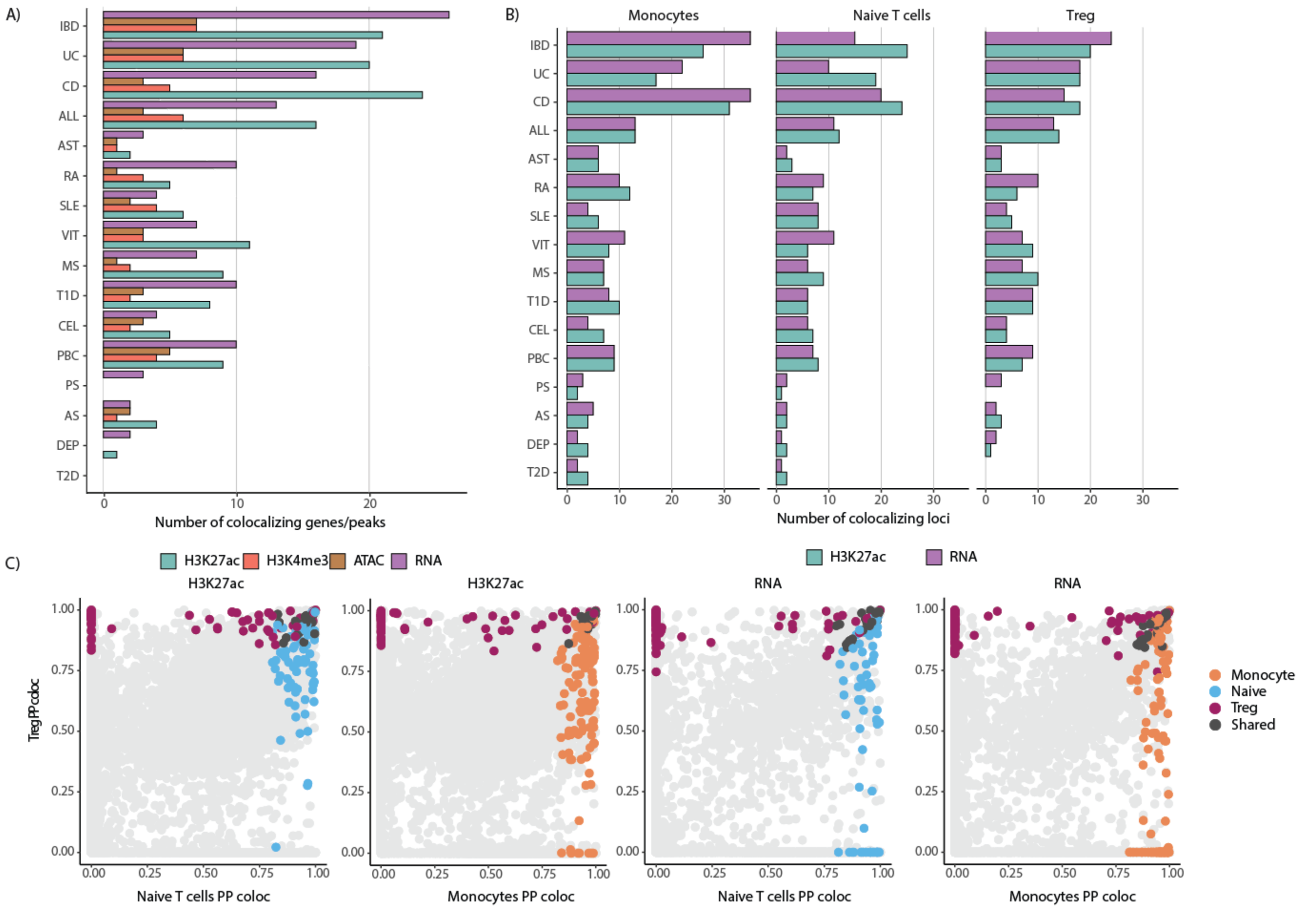

Supplementary Figure 5. Colocalization analysis between Treg QTLs and immune GWAS studies. A) Number of genes or peaks colocalizing with selected immune GWAS studies across the four genomic assays in Tregs. **B)** Comparison of number of GWAS loci colocalizing with eQTLs and actQTLs detected in naive T cells, monocytes and Tregs. **C)** Posterior probability (PP) of coloc for the same genes (eQTLs) or peaks (actQTLs) across all immune GWAS studies between Tregs and naive T cells or monocytes. Related to Figure 3.

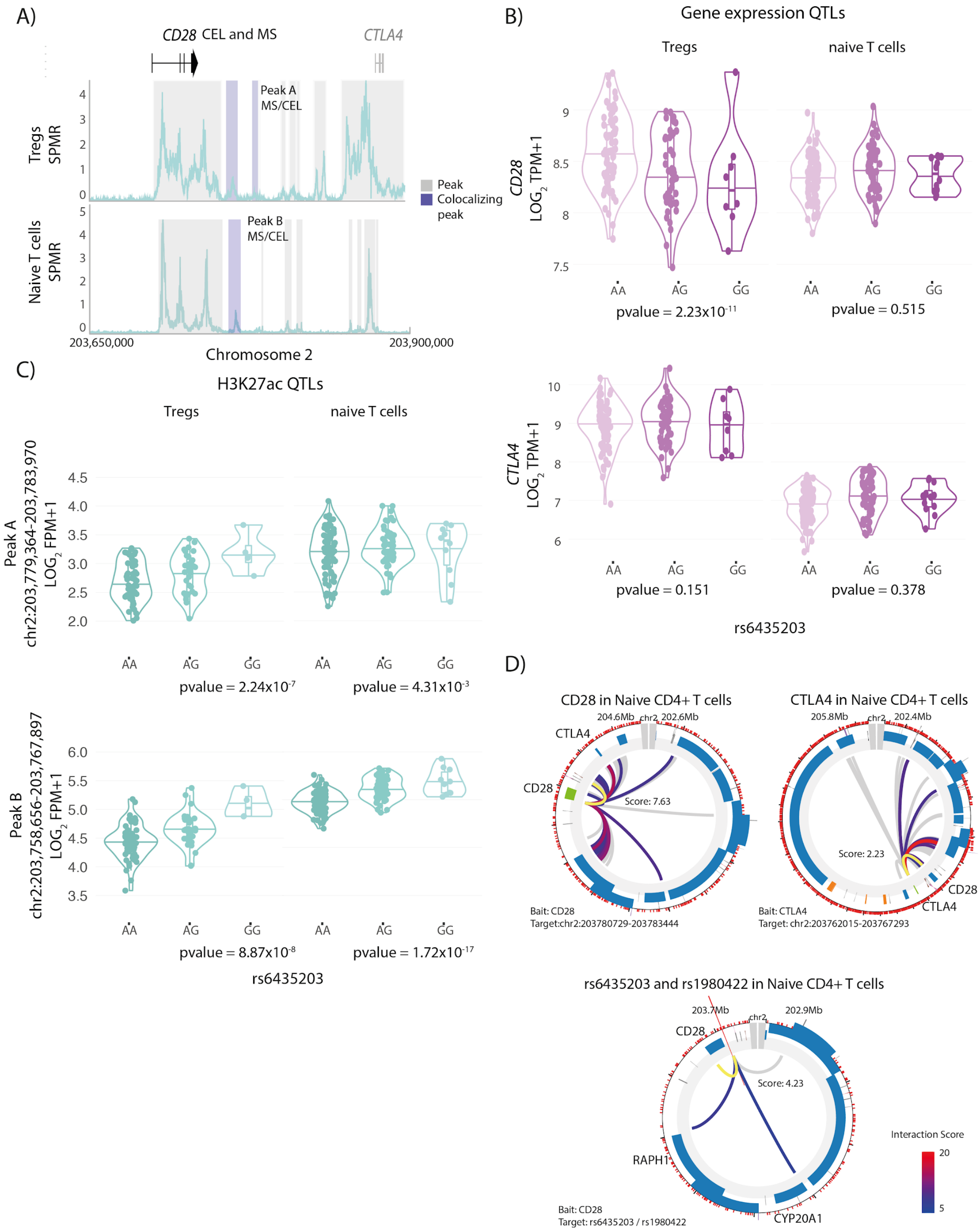

Supplementary Figure 6. Complex colocalization pattern of celiac disease and multiple sclerosis variants with cell type specific eQTLs and chromQTLs in naive and regulatory T cells at CD28/CTLA4 locus. A) Variants associated with CEL and MS, tagged by chr2:203,745,673 (rs1980422) and chr2:203,746,472 (rs6435203) respectively, are colocalizing with a *CD28* eQTL, with Peak A (an actQTL chr2:203,779,364-203,783,970) only present in Tregs and Peak B (an actQTL chr2:203,758,282-203,767,897) only present in naive T cells. **B)** *CD28* and *CTLA4* eQTLs in Tregs and naive T cells stratified by the MS associated rs6435203 genotype. The disease risk allele for MS, (rs6435203-A, major allele), resulted in increased levels of *CD28* expression, while the risk allele for CEL, chr2:203,746,472 (rs1980422-C, minor allele) resulted in increased the levels of *CD28* mRNA (both variants are

highly linked, R2 = 0.88). The levels of *CTLA4* mRNA were not affected by genotype. **C)** Peak A and Peak B actQTLs in Tregs and naive T cells stratified by MS associated rs6435203 genotype. The risk allele for MS decreased the H3K27ac levels while the risk allele for CEL resulted in increased acetylation. Rs6435203 was a significant actQTL for peaks A and B in Tregs, but this variant colocalized only with peak B in naive T cells (a similar colocalization pattern was observed for the CEL associated variant, rs1980422). **D)** Promoter capture Hi-C plots of the interaction scores for the *CD28*, *CTLA4* and rs1980422 or rs6435203 loci in naive CD4+ T cells published in Javierre *et al*. 2016 [1] (generated using Capture HiC Plotter online tool, https://www.chicp.org/chicp/). The Peak A region interacts with the *CD28* promoter, while the Peak B region interacts with the *CTLA4* promoter. Both SNPs interacted with the promoter of *CD28* in naive and total CD4+ T cells. CEL variant (rs1980422) was also an eQTL for *CTLA4* in CD4+, CD8+ cells and in testis [2,3], and interacted with the promoter of *CTLA4* in total CD4+ cells [1]. CTLA4, which inhibits T cell mediated immune responses by outcompeting CD28 for ligand binding [4]. Coordinates correspond to the GRCh38 build. Bait and target coordinates and interaction scores for specific connections (yellow). Related to Figure 4.

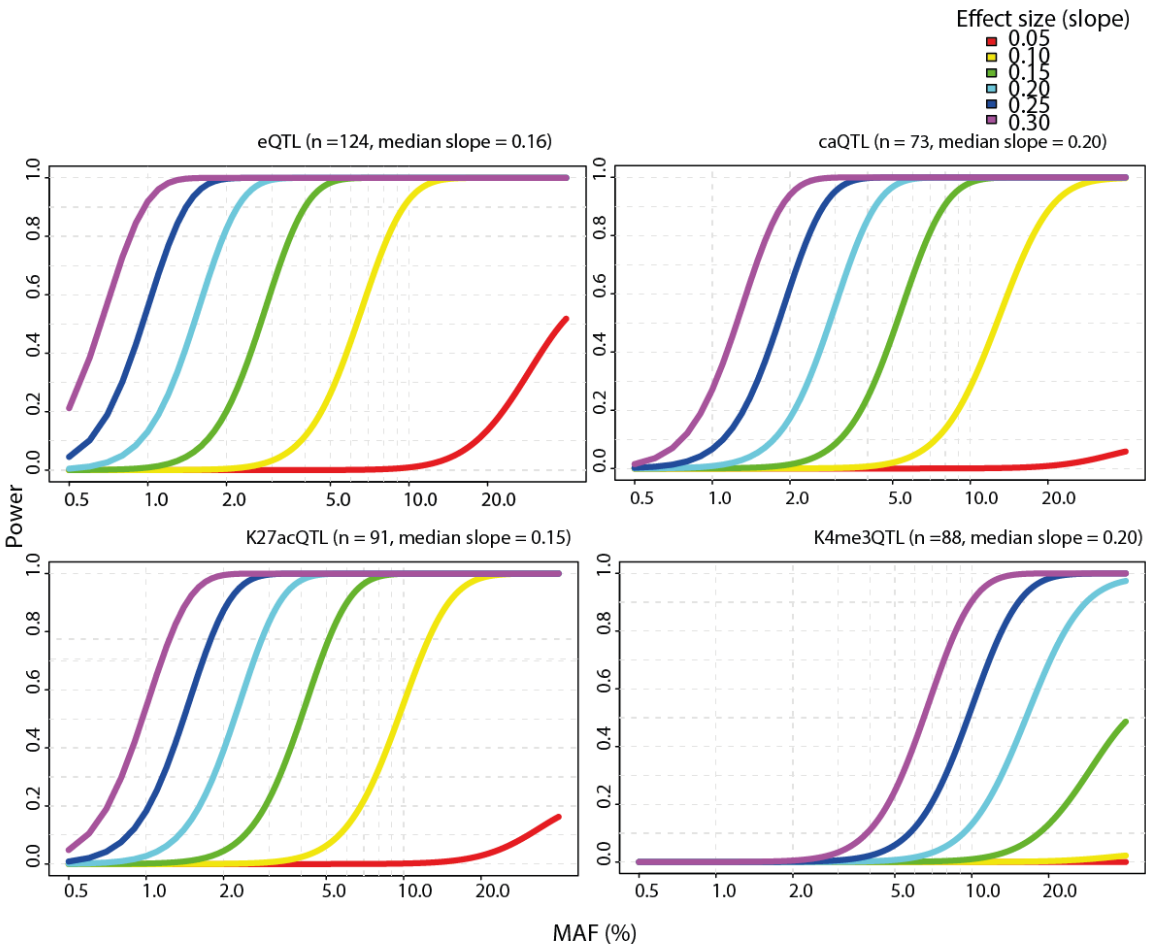

Supplementary Figure 7. Power calculations for QTL discovery using different functional genomic assays. Power was calculated for each assay using the powerEQTL package. Calculations are adjusted by sample size, number of tested SNPs, average reads per feature per assay. Power (y-axis) is plotted against minor allele frequency (MAF; x-axis). Different coloured lines correspond to different linear model slopes. Related to STAR Methods: Quantitative trait locus mapping (QTLs)

Supplementary References

1. Javierre BM, Burren OS, Wilder SP, Kreuzhuber R, Hill SM, Sewitz S, et al. Lineage-Specific Genome Architecture Links Enhancers and Non-coding Disease Variants to Target Gene Promoters. Cell. 2016 Nov 17;167(5):1369–84.e19.

2. Kasela S, Kisand K, Tserel L, Kaleviste E, Remm A, Fischer K, et al. Pathogenic implications for autoimmune mechanisms derived by comparative eQTL analysis of CD4+ versus CD8+ T cells. PLoS Genet. 2017 Mar;13(3):e1006643.

3. Gamazon ER, Segrè AV, van de Bunt M, Wen X, Xi HS, Hormozdiari F, et al. Using an atlas of gene regulation across 44 human tissues to inform complex disease- and trait-associated variation. Nat Genet. 2018 Jul 1;50(7):956–67.

4. Qureshi OS, Zheng Y, Nakamura K, Attridge K, Manzotti C, Schmidt EM, et al. Trans-endocytosis of CD80 and CD86: a molecular basis for the cell-extrinsic function of

CTLA-4. Science. 2011 Apr 29;332(6029):600–3.
